# A Repetitive DNA Element Regulates Expression of the *Helicobacter pylori* Sialic Acid Binding Adhesin by a Rheostat-like Mechanism

**DOI:** 10.1371/journal.ppat.1004234

**Published:** 2014-07-03

**Authors:** Anna Åberg, Pär Gideonsson, Anna Vallström, Annelie Olofsson, Carina Öhman, Lena Rakhimova, Thomas Borén, Lars Engstrand, Kristoffer Brännström, Anna Arnqvist

**Affiliations:** 1 Dept of Medical Biochemistry and Biophysics, Umeå University, Umeå, Sweden; 2 Dept of Microbiology, Tumor and Cell Biology, Karolinska Institute, Solna, Sweden; Fred Hutchinson Cancer Research Center, United States of America

## Abstract

During persistent infection, optimal expression of bacterial factors is required to match the ever-changing host environment. The gastric pathogen *Helicobacter pylori* has a large set of simple sequence repeats (SSR), which constitute contingency loci. Through a slipped strand mispairing mechanism, the SSRs generate heterogeneous populations that facilitate adaptation. Here, we present a model that explains, in molecular terms, how an intergenically located T-tract, via slipped strand mispairing, operates with a rheostat-like function, to fine-tune activity of the promoter that drives expression of the sialic acid binding adhesin, SabA. Using T-tract variants, in an isogenic strain background, we show that the length of the T-tract generates multiphasic output from the *sabA* promoter. Consequently, this alters the *H. pylori* binding to sialyl-Lewis x receptors on gastric mucosa. Fragment length analysis of post-infection isolated clones shows that the T-tract length is a highly variable feature in *H. pylori*. This mirrors the host-pathogen interplay, where the bacterium generates a set of clones from which the best-fit phenotypes are selected in the host. *In silico* and functional *in vitro* analyzes revealed that the length of the T-tract affects the local DNA structure and thereby binding of the RNA polymerase, through shifting of the axial alignment between the core promoter and UP-like elements. We identified additional genes in *H. pylori*, with T- or A-tracts positioned similar to that of *sabA*, and show that variations in the tract length likewise acted as rheostats to modulate cognate promoter output. Thus, we propose that this generally applicable mechanism, mediated by promoter-proximal SSRs, provides an alternative mechanism for transcriptional regulation in bacteria, such as *H. pylori*, which possesses a limited repertoire of classical trans-acting regulatory factors.

## Author Summary

During persistent *H. pylori* infection, the local gastric milieu is constantly altered by host responses and inflammation fluxes. As adhesion is crucial to maintain infection, appropriate adaptation of bacterial adherence properties is required to meet these environmental fluctuations. *H. pylori* uses the SabA protein to bind glycan receptors present on inflamed stomach mucosa. SabA expression can be turned on or off via known genetic mechanisms; however, how fine-tuning of SabA expression occurs to match changes in receptor levels is still unknown. The *H. pylori* genome encodes few trans-acting regulators but has numerous simple sequence repeats (SSR), *i.e.* hypermutable DNA segments. Here, we have deciphered a mechanism where a T-repeat tract, located in the *sabA* promoter region, affects SabA expression. The mechanism involves structural alterations of the promoter DNA that affects interaction of the RNA polymerase, without input from known trans-acting regulators. This mechanism is likely not unique for SabA or to *H. pylori*, but also applicable to other pathogens with high abundance of SSRs and limited set of transcription factors. Our findings contribute to understanding of the important bacterial-host interplay, and to mechanisms that generate heterogeneous populations of best-fit clones, *i.e.* stochastic switching.

## Introduction

A key factor for bacterial pathogens to establish and maintain a persistent infection is the adaptation to host responses and to microenvironmental alterations that occur during pathogenesis. Both stochastic and regulated processes can affect gene expression, and contribute to population heterogeneity. From the plethora of clones, best-fit phenotypes arise to match the current environmental demands. Population heterogeneity can be achieved by epigenetic events, such as DNA methylations; or strictly genetic mechanisms, such as reversible phase variation *e.g.* homologous recombination or slipped strand mispairing (SSM) of simple sequence repeats (SSRs) [1], [2]. SSRs create so-called contingency loci, *i.e.* hypermutable DNA that mediates stochastic genotypic switching, and these regions are often evolutionary conserved [3], [4]. The role of SSM in regulation of mRNA levels and protein expression is determined by the genetic location of the SSR. Intragenic SSRs cause biphasic translational control and turn protein expression on or off, while intergenic SSRs, may result in altered mRNA levels by different mechanisms [5], [6].

*Helicobacter pylori* infects the human stomach and if left untreated causes chronic gastritis that potentially leads to peptic ulcer disease and gastric cancer [7]–[9]. Adhesion is a prerequisite to establish persistent infection. The two dominating carbohydrates targeted by *H. pylori* in the gastric mucosa are the ABO/Leb blood group and the sialyl Lewis x/a (sLex/sLea) antigens [10]–[14]. In healthy mucosa the ABO/Leb antigens predominate, whereas the sLex/sLea antigens dominate the inflamed mucosa. *H. pylori* binds the ABO/Leb-receptors via the blood group antigen binding adhesin BabA, and the sLex/sLea-receptors via the sialic acid binding adhesin SabA. Since the human stomach glycosylation pattern constantly changes, *H. pylori* needs to adapt its adherence properties accordingly. Expression can efficiently be switched on or off via homologous recombination, or via SSM events [13], [15]–[18]. The protein expression of the BabA and SabA adhesins also varies between strains [15], [16], [19], [20].

Detailed studies of adhesin expression regulation in *H. pylori* are scarce. In other eubacteria, RNA polymerase sigma (σ) factors and transcriptional regulators control gene expression at the mRNA level. These likely play a diminished role in *H. pylori*, as only three σ-factors (σ^80^, σ^54^ and σ^28^) and few classical trans-acting regulators are present [21]–[23]. Thus, fine-tuning of mRNA levels in *H. pylori* likely involve alternative processes. *H. pylori*, like other bacteria with small genomes, has a high content of SSRs, primarily in genes encoding outer membrane proteins *e.g. alpA*, *alpB*, *babA*, *babB*, *sabA* and *sabB* [24]–[26]. In *H. pylori*, the impact of SSRs is probably further accentuated by the lack of mismatch repair systems and proof reading deficiency of the DNA polymerase I [27], [28]. In this context, SSM can rapidly create a large pool of heterogeneous clones and not surprisingly, *H. pylori* has an extremely high intraspecies genetic variability [29]–[32].

A cytosine-thymine dinucleotide (CT) repeat tract in the 5′-end of the *sabA* coding sequence (CDS) causes translational frameshifts and on/off phase variation [13], [15]. Additionally, a thymine (T) nucleotide repeat tract is found adjacent to the *sabA* −35 promoter element. The length of this T-tract varies between strains and such length variations have been suggested to influence *sabA* expression [33], [34]; however, the functional mechanism of how the T-tract regulates transcription remains to be elucidated. In this paper, we present data illustrating that the T-tract length, in clones isolated post-infection from different local gastric environments, is variable *in vivo*. We also demonstrate that the T-tract length controls *sabA* transcription initiation, and thus SabA expression and functional sLex-receptor binding to gastric mucosa, in a multiphasic manner by affecting binding of the RNA polymerase. We describe in molecular terms how the T-tract length influences the local DNA structure, by changing the axial alignment between the core promoter and UP-like elements, thereby affecting interaction of the RNA polymerase α-subunits to the *sabA* promoter. In addition, we provide evidence that a similar mechanism controls multiple loci in *H. pylori*. Therefore, we propose a generally applicable model in which T- or A-tracts located adjacent to −35 promoter elements act by a rheostat-like mechanism, to control transcription initiation in *H. pylori*.

## Results and Discussion

### The T-tract fine-tunes *sabA* expression and consequently binding to the sialyl Lewis x receptor

**Figure 1:**
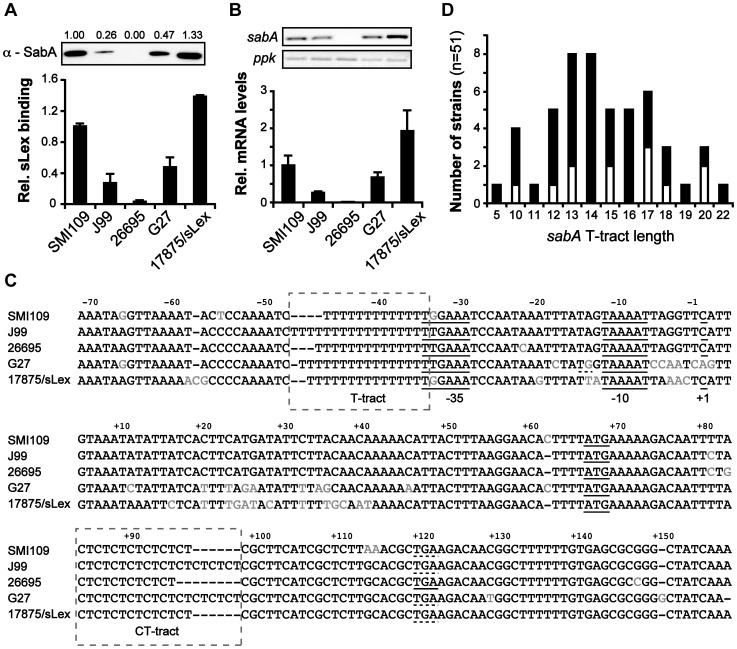
Interstrain variation of *sabA* mRNA levels, SabA protein expression and functional sLex-receptor binding. A) Analysis of SabA expression and sLex-receptor binding activity in a set of five *H. pylori* strains. Image shows one representative immunoblot analysis with α-SabA antibodies and the numbers above represents SabA expression quantification, with expression in strain SMI109 set to 1. Equal amounts of crude protein extracts were loaded in each lane (Fig. S10A). The graph shows binding to soluble ^125^I-sLex-receptor conjugate of the same strains as analyzed in the immunoblot. Bacteria were grown on plate as described in Materials and Methods prior to the analysis. Average and standard deviations are calculated from at least two independent experiments and duplicate samples/analysis of each strain. B) RT-qPCR analysis of *sabA* mRNA levels in the same set of strains as in Fig. 1A. The *sabA* mRNA levels were normalized to a set of reference genes and data is presented as relative, with the levels in strain SMI109 set to 1. Images show one representative semi-quantitative PCR analysis, using the same primers as in the RT-qPCR analysis; *sabA*-2 and *ppk*-2. C) Sequence comparison of the P*sabA* region (−71 to +158, relative to the transcriptional start site) between different *H. pylori* strains. The +1 transcriptional start sites, as determined by primer extension and 5′-RACE, and the predicted −10 and −35 promoter elements, are underlined. Differences in nucleotide sequences are shown in grey color. The regions containing the T-tract and CT-repeats are boxed. The stop codon (TGA) that results in a truncated SabA protein in the CT_6_-off strain 26695 is also underlined. A more extensive comparison, of 44 P*sabA* sequences, is shown in Fig. S2. D) Distribution of T-tract lengths in the *sabA* promoter (P*sabA*) of 51 sequenced *H. pylori* strains. Black represents number of analyzed genome-sequenced strains, whereas white represents the number of strains where the sequence of the *sabA* locus was obtained after conventional PCR amplification.

It was previously shown that expression of SabA varies among different clinical isolates and that expression levels match the binding activity to the cognate sialyl Lewis x (sLex) receptor [15], [19], [20], [35]. In this study, we set out to scrutinize determinants that cause these differences. A set of five *H. pylori* strains, representing numerous geographical origins and isolated from patients with different disease symptoms (described in Table 1), were chosen for the analysis. SMI109 (Sweden, GC), J99 (USA, DU), G27 (Italy, GA), 17875/sLex (Australia, GA) and 26695 (UK, GA) were assayed for SabA protein expression by immune-detection, and for receptor binding activity by RadioImmunoAssay (RIA) using ^125^I-sLex-receptor conjugates. As expected strain 26695, with a predicted frameshift in the *sabA* CT-tract, did not express any SabA protein nor could it bind to sLex-receptor conjugates (Fig. 1A and 1C). Strains SMI109 and 17875/sLex displayed highest SabA expression and accordingly cognate sLex-receptor binding activity, whereas strains J99 and G27 displayed intermediate levels of both (Fig. 1A). These results confirmed the significant variation of SabA expression between strains and the link between protein expression and receptor binding activity.

**Table 1:** Strains and plasmids used in this study.

Name	Characteristics	Reference
***Strains***		
DH5α	*E. coli*	Laboratory stock
AAG1	*E. coli*, MG1655 Δ*lacZ*	[89]
J99	*H. pylori* clinical isolate, USA, Duodenal ulcer	[91]
J99^StrR^	Streptomycin resistant variant of J99, mouse-adapted	This study
26695	*H. pylori* clinical isolate, UK, Gastritis	[54]
G27	*H. pylori* clinical isolate, Italy, Gastritis	[92]
17875/sLex	*H. pylori* clinical isolate, Australia, Gastritis	[93]
SMI109	*H. pylori* clinical isolate, Sweden, Gastric cancer	[13]
SMI109 Δ*sabA*	Δ*sabA*::*kan* variant of SMI109	This study
SMI109 Δ*sabA*::*rpsLCAT*	*sabA* promoter in SMI109 replaced by *rpsLCAT* cassette	This study
SMI109 T-variants	Constructed variants of SMI109 with different repeat tract length in *sabA* promoter; T_1_–T_21_ and A_13_, C_13_	This study
SMI109 *pyrG*::*lacZ*	SMI109 harboring pAAG202 on the chromosome, Km^R^	This study
SMI109 *pyrGΔ5*::*lacZ*	SMI109 harboring pAAG203 on the chromosome, Km^R^	This study
SMI109 *hp_0350*::*lacZ*	SMI109 harboring pAAG204 on the chromosome, Km^R^	This study
SMI109 *hp_0350Δ5*::*lacZ*	SMI109 harboring pAAG205 on the chromosome, Km^R^	This study
SMI109 Δ*hup*	Δ*hup*::*kan* variant of SMI109	This study
SMI109 Δ*napA*	Δ*napA*::*kan* variant of SMI109	This study
***Plasmids***		
pCR TOPO	General cloning vector, Cb^R^	Life Technologies
pUC19	General cloning vector, Cb^R^	[94]
pRZ5202	*lacZ* promoter fusion vector for *E. coli*, Cb^R^	[95]
pBW	*lacZ* promoter fusion vector for *H. pylori*, Km^R^	[96]
pAAG132	*sabA* promoter from 26695 cloned in pRZ5202, Cb^R^	This study
pAAG134	*sabA* promoter from J99 cloned in pRZ5202, Cb^R^	This study
pAAG135	*sabA* promoter from G27 cloned in pRZ5202, Cb^R^	This study
pAAG136	*sabA* promoter from 17875/sLex cloned in pRZ5202, Cb^R^	This study
pAAG107	*sabA* promoter from SMI109 cloned in pRZ5202, Cb^R^	This study
pAAGXX*	Variants of pAAG107 with different length of repeat tract in *sabA* promoter; T_1_–T_21_ and A_13_, C_13_	This study
pAAG198-201	Δ_46_ variants of P*sabA*::*lacZ*, with different length of repeat tract in *sabA* promoter; T_9_, T_13_, T_18_ and A_13_	This study
pAAG206-208	Scrambled UP-like elements of P*sabA*::*lacZ*	This study
pAAG202	*pyrG* promoter from SMI109 cloned in pBW, Km^R^	This study
pAAG203	*pyrG* promoter from SMI109, lacking 5T's, cloned in pBW, Km^R^	This study
pAAG204	*hp_0350* promoter from SMI109 cloned in pBW, Km^R^	This study
pAAG205	*hp_0350* promoter from SMI109, lacking 5A's, cloned in pBW, Km^R^	This study
pAAG178	Δ*hup*::*kan* construct cloned in pUC19, Km^R^, Cb^R^	This study
pBlue+Δ*napA*	Δ*napA*::*kan* construct cloned in pBlueScript, Km^R^, Cb^R^	[78]
pKD4	Used as template for kanamycin resistance cassette	[97]

* Several plasmids with *sabA*::*lacZ* transcriptional fusion, with different length of the T-tract, were used.

To establish if mRNA levels were related to the SabA protein expression, we analyzed *sabA* mRNA levels with RT-qPCR in the corresponding *H. pylori* strains. A clear correlation was observed (Fig. 1B). We also generated transcriptional *lacZ* reporter fusions of the *sabA* promoter (P*sabA*) from the different strains (Fig. S1A) and found transcriptional initiation to vary when measuring promoter activity by β-galactosidase assay in *E. coli* (Fig. S1B). However, the promoter activities did not correlate with the mRNA levels or SabA protein expression found in the different *H. pylori* strains. For example, activity of the P*sabA* from strain 26695 was comparatively high, considering that this strain did not express any detectable cognate *sabA* mRNA (compare Fig. 1B and S1B). This is likely explained by the correlation between transcriptional and translational processes in *H. pylori* recently shown [36] and illustrates that downstream effectors, like mRNA stability or *H. pylori* specific factors, are essential for absolute mRNA levels. Further, this emphasizes the importance of studying expression in an isogenic strain background.

Sequencing of the P*sabA* region from the different strains revealed scarce nucleotide variations scattered across the promoter. Some exchanges in the −10 and −35 promoter elements were observed, as well as length variations in the T-tract located adjacent to the −35 element (Fig. 1C). *sabA* from strain G27 shows a nearly perfect extended −10 promoter element (TGnTAAAAT vs TGnTATAAT in *E. coli*), which explains the high promoter activity observed with *lacZ* fusions in *E. coli* for this promoter (Fig. S1B). Analysis of a larger set of P*sabA* sequences revealed unusual high homology, except for a major discrepancy in the length of the T-tract (Fig. S2). If the T-tract could play a role in regulating *sabA* expression, we reasoned that the T-tract length might vary extensively between *H. pylori* strains to match the present sLex-receptor availability in each infected individual. Therefore, we compared the T-tract length of forty-nine published *H. pylori* genome sequences and sequenced the P*sabA* of twelve additional strains. In total, we found fifty-one strains to encode a *sabA* gene. As assumed, there was a wide distribution of T-tract lengths, ranging from T_5_ to T_22_, where T_13_ to T_17_ being the most common variants (Fig. 1D and Table S1). In a collection of 115 clinical Taiwanese isolates, Kao *et al* [34] showed that the T-tract length varied from T_10_ to T_28_, with the most common variants being T_14_ to T_19_. In further support of the idea for individual selection, we could not find any obvious geographic correlations of T-tract lengths, as exemplified by the extensive T-tract variation (T_5_ to T_16_) in the different Peruvian strains analyzed (Cuz20, PeCan4, PeCan18, Puno120, Puno135, Sat464, Shi112, Shi169, Shi417, Shi470, SJM180; Table S1).

Since a number of factors affect transcription, at different regulatory levels, we generated transcriptional P*sabA*::*lacZ* reporter fusions to determine if the T-tract length could impact transcription initiation in a given strain. The P*sabA*::*lacZ* fusions were based on the P*sabA* of the SabA high-expressing strain SMI109, but with varying T-tract lengths comparable to the T-length distribution described in the preceding section (Fig. 1D, T_1_ to T_21_). The β-galactosidase assays were performed in *E. coli*. First, we used 5′ rapid amplification of cDNA ends (5′-RACE) and determined the transcriptional start site of *sabA* in SMI109 to be located at a cytosine, 66 nt upstream of ATG, the same transcriptional start site as previously published for J99 [34]. We also verified that an identical transcriptional start site was used in *E. coli* as in *H. pylori* by primer extension analysis (data not shown). The β-galactosidase assays revealed that the promoter activity of the P*sabA*::*lacZ* fusions with varying T-tract length was gradually multiphasic: high in T_5_, low in T_9_, intermediate in the T_13_ (wt) and high in T_18_ (Fig. S1C).

**Figure 2:**
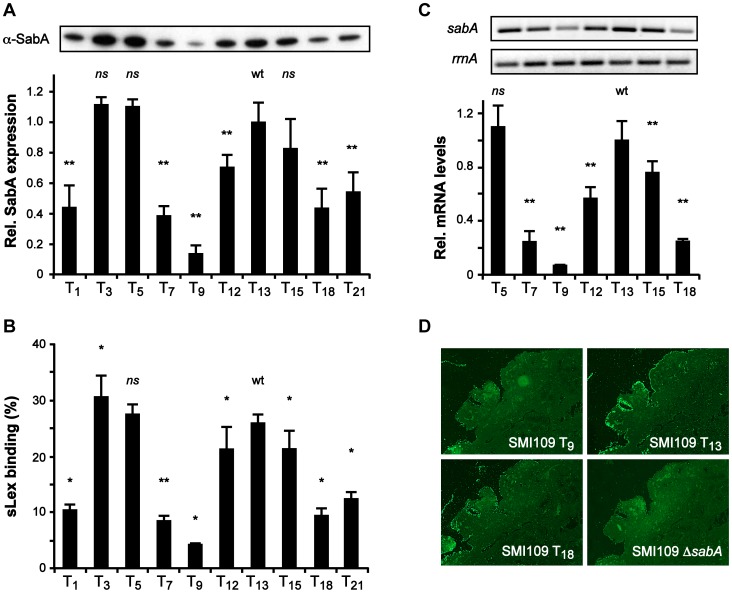
The T-tract length alters sLex-receptor binding activity by affecting *sabA* mRNA levels in *H. pylori*. A) SabA protein expression analysis in variants of SMI109 harboring different T-tract lengths. Image shows one representative immunoblot with α-SabA antibodies used for the quantification. Expression levels were normalized to expression of the AlpB protein before comparison (Fig. S10B). Data are presented in the bar diagram, as described in Fig. 1A, with the expression in the T_13_ (wt) variant set to 1. Stars indicate significant differences from T_13_-variant, * *p*<0.05, ** *p*<0.01, ns = non significant. B) Binding to soluble ^125^I-sLex-receptor conjugates of the same set of T-variants as in Fig. 2A. The data are presented as in Fig. 1A, with the binding of the T_13_-variant set to 1. Stars mark significant differences from T_13_-variant, see Fig. 2A. C) RT-qPCR analysis of *sabA* mRNA levels in T-variants of SMI109. Data are presented as in Fig. 1B, with the mRNA levels in the T_13_-variant (wt) set to 1. The upper images show result from one semi-quantitative PCR analysis using primers for *sabA*-1 and *rrnA*-2. D) Binding of FITC-labeled SMI109 T-variants (T_9_, T_13_ and T_18_) to human gastric tissue sections. SMI109 Δ*sabA* mutant was included as a negative control. Images were taken with 100× magnification. For all analyses in Fig. 2, bacteria were grown on plate prior to the experiment, as described in Materials and Methods.

These results suggested that the T-tract length affects promoter activity. To further analyze this in *H. pylori*, we decided to explore the role of the T-tract in otherwise isogenic variants of strain SMI109, with P*sabA* T-tracts spanning from T_1_ to T_21_, the same set of T-variants that were analyzed in *E. coli*. Such variants were obtained by exchanging the P*sabA* region in SMI109 using a method involving contraselection in combination with *in vitro* mutagenesis (see Materials and Methods). First, SabA protein expression and sLex-receptor binding activity were analyzed in these variants. This revealed an even more pronounced multiphasic appearance than in *E. coli*, although in *H. pylori* the T_13_ variant exhibited high and the T_18_ variant intermediate protein expression and sLex-receptor binding activity (Fig. 2A–B). We also determined the *sabA* mRNA levels in the T-tract variants T_5_ to T_18_ with RT-qPCR (Fig. 2C). The mRNA level was likewise gradually multiphasic and closely correlated to the protein expression and receptor activity (Fig. 2). Interestingly, the max/min protein and mRNA levels were observed with T-tract length intervals of approximately ten base pairs, the same distance as one turn of the DNA helix.

To mimic *H. pylori* adhesion during *in vivo* conditions and the presentation of the sLex-receptor on epithelial cells, we analyzed SabA-mediated adhesion to human gastric tissue sections. Gastric tissue sections were probed with fluorescently labeled *H. pylori* of varying T-tract lengths, which displayed different sLex binding activity. The SabA high-expressing T_13_ variant clearly exhibited more binding to the tissue sections than the low-expressing T_9_ and intermediate-expressing T_18_ variants (Fig. 2D). In contrast, neuraminidase-treated mucosa, where the sialic acid moieties had been removed, showed only background binding (data not shown). Likewise, a Δ*sabA* mutant derivative of SMI109 exhibited no binding to the tissue sections (Fig. 2D). Thus, our results demonstrate that variations in the T-tract length, in otherwise isogenic strains, affect P*sabA* activity. This induces multiphasic alterations of *sabA* mRNA levels and thereby SabA protein expression levels which adjust the sLex-receptor binding activity and binding to human gastric mucosa.

### Variations in T-tract length during infection

At the *H. pylori* infection site, a local inflammation develops as a result of the host cell responses and release of effector molecules. Earlier experimental *H. pylori* infections in Rhesus monkeys showed variable reciprocal changes of both fucosylated ABO/Leb and sialylated sLex/a receptor expression during infection [14], [37], [38]. These alterations need to be accompanied by changes of BabA and SabA adhesin expression to maintain infection. Consequently, there is a delicate balance to cycle between non-adherent and adherent states, and to adjust expression levels at the adherent state. We have previously shown that Mongolian gerbils infected with *H. pylori* strains, expressing different levels of BabA adhesin, display alterations in the infection load and host cell responses, as well as phenotype modifications of the infected strain [39]. This scenario likely mirrors the host-pathogen interplay and post-experimental output clones are thus the result of a host-biopanning procedure that selects for clones with best-fit phenotype, *i.e.* stochastic switching.

Since our results suggest that the T-tract length ultimately controls sLex-receptor binding activity, selection for certain T-tract variants likely occurs as SabA expression is optimized to the receptor availability in a respective host. As outlined in the preceding section, we propose that the T-tract length is variable over time and under different selection conditions. To study T-tract length variations, and the corresponding functional alteration in sLex-receptor binding over time, we subjected strain SMI109 to a series of *in vitro* passages for three months. sLex-receptor binding post-passages revealed sub-populations that displayed lower sLex-receptor binding activity (sLex-low) relative to the other sub-populations (sLex-high, data not shown). Single clones were isolated from both sub-populations, and the cognate P*sabA* regions were sequenced. This analysis revealed that the sLex-high clones had a T_13_ tract (wt), while the sLex-low clones had T_12_ tract in the P*sabA* region (Fig. S3A). A similar experiment was also performed with strain J99 and comparable results were observed. Here the sLex-high clones had a T_18_ tract (wt), while the sLex-low clones had T_19_ tract in the P*sabA* region (Fig. S3B).

**Figure 3:**
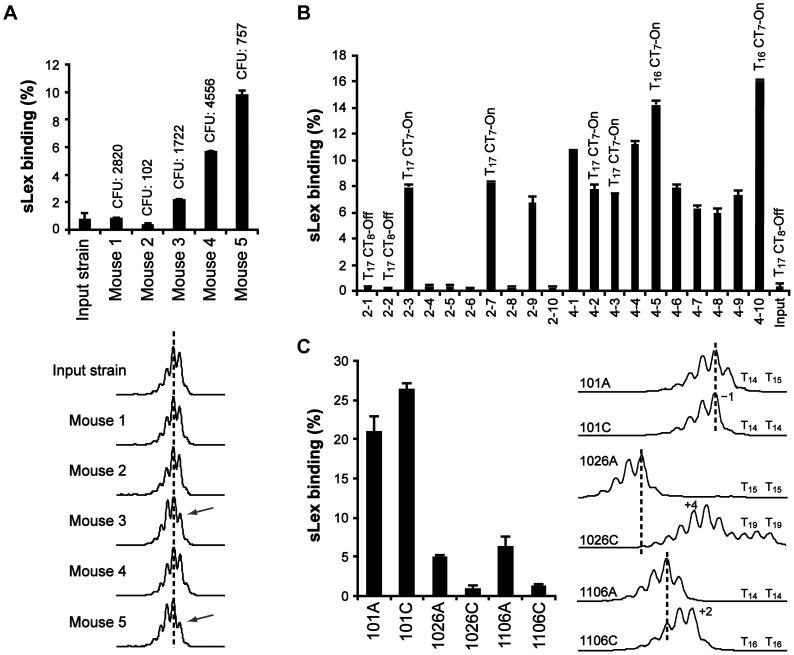
The T-tract length is variable, both in a mouse model and in the human stomach. A) Analysis of bacterial output pools isolated two months post-infection from FVB/N mice. Binding to soluble ^125^I-sLex-receptor conjugates is shown in the bar diagram. Values above the bars show the infectious load in each mouse (colony forming units, CFU). Bottom curves show the corresponding FLA-spectra after PCR-amplification of the P*sabA* region, using genomic DNA isolated from the different output pools as template, including the input strain. Dotted lines serve as length reference for comparison. The arrows mark the FLA peak observed to decrease in output pools of mouse 3 and 5, relative to input strain. B) Binding to ^125^I-sLex-receptor conjugates of ten independent clones isolated from the bacterial output-pools of mouse 2 and 4, respectively. The T- and CT-tract lengths of a representative set of clones, as determined by sequencing, are shown above the bars. CT_7_-On = SabA CDS in-frame, CT_8_-off = SabA CDS out of frame. C) Analysis of bacterial output pools, isolated from the antrum (A) and the corpus (C) regions of the stomach, of three Swedish patients. Binding to soluble ^125^I-sLex-receptor conjugates of the output pools is shown in the bar diagram, and the corresponding FLA-spectra are shown to the right. The T-tract lengths, of two clones from each bacterial pool, are shown next to the FLA-spectra.

Next, we used a mouse-adapted sLex-negative clone of strain J99^StrR^ (*sabA* T_17_ and CT_8_-off) to infect five Lewis b (Leb) transgenic FVB/N mice, since we did not succeed to infect mice with strain SMI109 [40]. After two months, the mice were terminated and the output bacteria (pools) were scored for recovery of sLex-receptor binding activity. Bacterial pools from three out of five mice had changed their adherence phenotype and were now positive for sLex-receptor binding (Fig. 3A, bar diagram). Sequencing of output pools often generates mixed peaks, likely due to population heterogeneity and loss of signal after repetitive DNA tracts. Therefore, to estimate changes in T- and CT-tract lengths, we used fragment length analysis (FLA) to discriminate length variations of PCR-amplicons, down to one nucleotide. Since FLA of repetitive DNA gives rise to additional “stutter” peaks [41], we obtained reference spectra of genomic DNA isolated from the otherwise isogenic T_18_ or T_19_ variants of SMI109, and correlated our results to these (Fig. S4). To confirm that changes in the CT-tract length caused the alterations in on/off sLex-receptor binding appearance of the output pools, we ran FLA with primers including part of the *sabA* coding sequence (CDS). This was exemplified by using genomic DNA isolated from one sLex-negative output pool (mouse 2) and one sLex-positive output pool (mouse 4), and also the sLex-negative input strain as reference. As expected, our analysis revealed two nucleotides shorter PCR-amplicons from output pools of mouse 4, corresponding to a change in the CT-tract length from eight to seven repeats, thereby placing translation in the right open reading frame. The PCR-amplicons of output pools of mouse 2 displayed the same length as the input strain (data not shown).

In order to only analyze changes in the T-tract length, we ran FLA with primers excluding the CT-tract. The results showed that bacterial pools from mouse 3 and 5 had a left-shifted curve pattern indicative of a larger subpopulation with one nucleotide shorter T-tract, which contrasts the unchanged curve pattern in pools from mouse 1, 2 and 4 (Fig. 3A, bottom curves). However, to determine if the FLA was limited in detecting smaller population changes in T- and CT-tract length, we next analyzed ten random clones from two distinctly different output pools not shifted in the FLA and with the lowest and highest CFU counts: the sLex-negative pool from mouse 2 and the sLex-positive pool from mouse 4 (Fig. 3A). First we determined the frequency of sLex-receptor binding in each population and as expected, all clones from mouse 4 showed binding (10/10 clones) while only 3/10 clones from mouse 2 showed sLex-receptor binding (Fig. 3B). The 5′ region of the *sabA* locus, in four representative clones from each bacterial pool, was sequenced and revealed that all sLex-negative clones were CT_8_-off, and all sLex-positive clones were CT_7_-On (Fig. 3B). Furthermore, among the sLex-positive clones, different binding activities were observed. The clones with highest sLex-receptor binding activity (4–5 and 4–10) had T_16_-tract in their P*sabA* region, in contrast to the other clones, which had T_17_ (Fig. 3B). FLA-spectra obtained for these clones corroborated the sequencing results (data not shown). Hence we concluded that although FLA gives a view of general population shifts in nucleotide repeat tract length, it is limited in detecting more discrete shifts as was seen in output pools from mouse 2 and 4. Overall, our results confirm the *in vitro* data obtained from SMI109 and J99, illustrating that small changes in T-tract length have major impact on sLex-receptor binding activity (Fig. 2B and Fig. S3).

To further investigate T-tract length alterations *in vivo*, we analyzed *H. pylori* output pools isolated from both antrum and corpus regions of the stomach, from three Swedish patients (here called: 101, 1026 and 1106). These bacterial pools were first analyzed for sLex-receptor binding activity. Bacterial pools from patient 101 showed high sLex-receptor binding, whereas lower binding was observed for the antrum-pools from patients 1026 and 1106. The sLex-receptor binding of the corpus-pools from patients 1026 and 1106 was barely detectable (Fig. 3C, bar diagram). We also isolated genomic DNA to obtain FLA-spectra of the 5′ region of the *sabA* locus from each pool (Fig. 3C, right curves). This revealed distinct curve patterns, in the bacterial pools from each stomach region and from each patient, respectively. FLA analysis of P*sabA* region alone, using primers excluding the CT-tract, showed similar results (data not shown). To confirm that the observed FLA alterations were a consequence of T-tract length variations, genomic DNA was isolated from two clones of each output pool, and the 5′-end of *sabA* locus was sequenced. The result showed similar T-tract length variations as detected by the FLA (Fig. 3C). Although it was not possible to make quantitative measurements using FLA technique (Fig. S4), it is a powerful tool to estimate variations in repeat tract lengths in heterogeneous populations.

Our data support the idea of individual selection since analysis of output clones, from infected mice as well as from distinct regions of the human stomach, revealed populations of heterogeneous sLex-receptor binding phenotypes and different T-tract lengths, which changed along the course of infection (Fig. 3). Interestingly, in one stomach we found a sLex-negative population (CT-off) that displayed a higher degree of T-tract length heterogeneity (Fig. 3C, sample 1026C). This suggests that without SabA-mediated adhesion, and corresponding host cell responses, there is no selection pressure directed against clones with certain SabA-expressing phenotypes and thus, all T-tract variants generated by SSM are preserved. It has been suggested that SSM frequencies could be affected by environmental stresses [6], however, how these signals are transduced to modulate switching rates are still unclear.

### The T-tract modifies RNA polymerase binding efficiency to the *sabA* promoter

SSRs located in intergenic regions have been reported to affect transcription by different mechanisms. SSRs positioned between the −10 and −35 promoter elements affect the docking of the RNAP sigma factor [42]–[45]. SSRs positioned upstream of the −35 element have been reported to affect binding of trans-acting factors and interaction with the RNAP [46]–[48]. SSRs located downstream of transcriptional start sites affect mRNA stability or binding of regulatory proteins [49], [50]. A recent study of a SSR in *H. pylori* shows that expression of the chemotaxis receptor *tlpB* is affected by a variable G-tract located downstream of the −10 element, via small RNA-mediated posttranscriptional regulation [51]. Depending of the length of the G-tract and interaction with the sRNA, expression of TlpB is either increased or decreased. Having ascertained that the length of our described T-tract affects *sabA* mRNA levels, we hypothesized that changes in RNA polymerase (RNAP) interaction with the P*sabA* DNA could underlie the observed variations in mRNA levels, since the T-tract is positioned adjacent to the −35 element.

**Figure 4:**
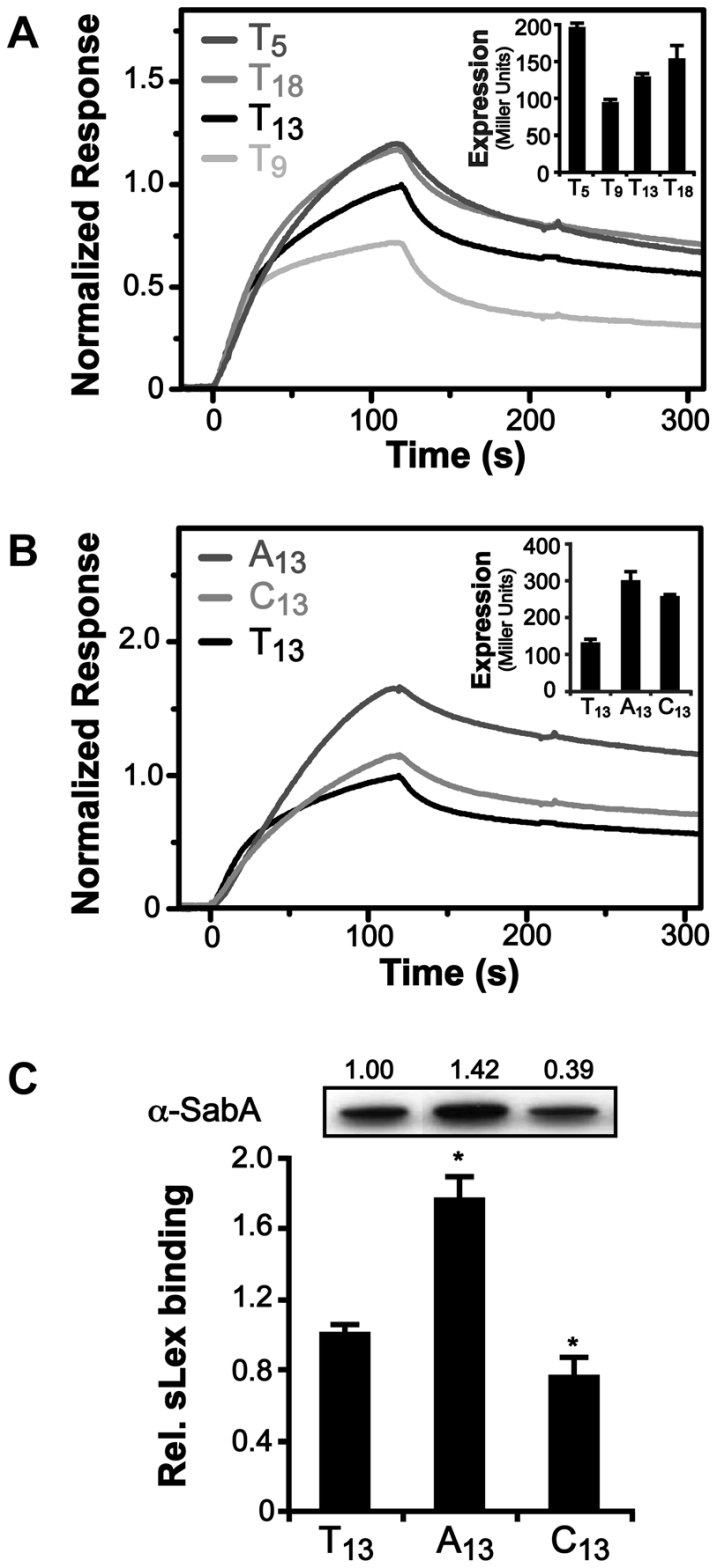
Binding of RNAP to P*sabA* DNA with varying tract length and nucleotide composition. A) Analysis of *E. coli* σ^70^-RNAP binding to P*sabA* DNA by Surface Plasmon Resonance (SPR). Sensorgrams show injection of the σ^70^-RNAP (20 nM) over chips with pre-bound biotinylated-P*sabA* (−166 to +74) DNA fragments, with different T-tract lengths (T_5_, T_9_, T_13_ or T_18_). Inlay shows promoter activity of the corresponding T-tract variants, assayed in *E. coli* using transcriptional P*sabA*::*lacZ* fusions as described in Fig. S1. B) SPR sensorgrams analyzed as described in 4A but with P*sabA* DNA fragments containing A_13_- or C_13_-tracts. Inlay shows promoter activity of the corresponding variants, assayed in *E. coli* using transcriptional P*sabA*::*lacZ* fusions, as in Fig. S1. C) Analysis of SabA expression and sLex-receptor binding activity of variants of SMI109 harboring A_13_- or C_13_-tracts in P*sabA*. The image shows one representative immunoblot with α-SabA antibodies, where numbers above represent relative expression with expression in the T_13_-variant set to 1. Bar diagram show binding to soluble ^125^I-sLex-receptor conjugate of the same set of variants as in the immunoblot. Samples were prepared as described in Fig. 1A. Statistical tests showed significant differences to the T_13_ (wt) variant (* = p<0.05).

The core promoter of P*sabA* (SMI109, TGGAAT-16 bp-TAAAAT) in strain SMI109 is similar to that of the *E. coli* housekeeping σ^70^ consensus binding site (TTGACA-17+/−1 bp-TATAAT), and highly homologous between different *H. pylori* strains (Fig. 1C and Fig. S2). No functional RNAP holoenzyme has yet been purified from *H. pylori*, however, the *E. coli* σ^70^-RNAP can bind and transcribe *H. pylori* promoters [52], [53]. Therefore, we tested binding of the *E. coli* σ^70^-RNAP to P*sabA* DNA fragments, using electrophoretic mobility shift assay (EMSA), and found it to interact strongly (Fig. S5, picture). No interaction was observed to *sabA* CDS DNA or when only the core RNAP was used (data not shown). When we analyzed σ^70^-RNAP binding to P*sabA* with varying T-tract lengths by EMSA, we could not detect differences in the amount of shifted DNA as the T-tract length was varied (Fig. S5, bar diagram). We instead decided to use high-resolution surface plasmon resonance (SPR) to obtain sensorgrams of σ^70^-RNAP binding to immobilized P*sabA* fragments with various T-tract lengths (T_5_, T_9_, T_13_, and T_18_). Now, we could clearly distinguish variations in binding strength to the P*sabA* fragments (Fig. 4A). As a control, the EMSA-inactive DNA fragment of *sabA* CDS showed no specific binding in the SPR analysis and was subtracted from each of the sensorgrams in Fig. 4. The results showed that σ^70^-RNAP displayed weakest binding to T_9_, but stronger binding to both T_5_ and T_18_, as compared to T_13_ (wt). The relative binding was comparable to the promoter activity of the various P*sabA* fragments, as measured by β-galactosidase assays using transcriptional fusions in *E. coli* (Fig. 4A inlay and Fig. S1C).

In order to investigate if the T-tract acts as a spacer, *i.e.* changing the distance and position of a binding site, we started by replacing the P*sabA* nucleotide content of the T-tract, without changing the length. The wt T_13_-tract was exchanged to A_13_ or C_13_ in the corresponding P*sabA*::*lacZ* fusion plasmids. Measurements of the promoter activities in *E. coli* showed that the P*sabA* activity, in both A_13_ and C_13_, increased relative the T_13_ variant (Fig. 4B, inlay). SPR analysis revealed higher binding of σ^70^-RNAP to the A_13_ then to the T_13_ variant, comparable to the P*sabA* activity (Fig. 4B). Conversely, for the C_13_ variant, the binding of σ^70^-RNAP was similar to that of the T_13_ variant. We also created isogenic A- and C-tract variants in strain SMI109 and found that replacement of T's to A's indeed gave higher SabA expression and sLex-receptor binding, whereas substitution of T's to C's gave slightly lower SabA expression, matching the SPR results (Fig. 4B–C). These results excluded that the T-tract merely acts as a spacer, as there were still variations in SabA expression levels, though the tract length was kept constant.

Thus, our results suggest that the T-tract modulates *sabA* transcription by changing the efficiency of RNAP binding. Nevertheless, *sabA* expression in *H. pylori* and *in vitro* RNAP binding did not exactly match. This could possibly be explained by alternative display of the RNAP binding site, caused by different organization of genomic DNA *in vivo* versus the shorter DNA fragments used in the *in vitro* SPR-analyses, or conceivably by additional unknown factors that impact *sabA* transcription in *H. pylori*. Another contributing factor could be the structural differences in the RNAP subunits between *E. coli* and *H. pylori*. The β- and β′-subunits have 45% identity to *E. coli* counterparts (RpoB and RpoC) but are expressed as a fused polypeptide in *H. pylori* [54]. This has been implied to facilitate the assembly of the holoenzyme [55] and to give a selective advantage for *H. pylori* fitness in the acidic human stomach [56]. The housekeeping sigma factor (σ^80^) from *H. pylori* has 32% identity to *E. coli* σ^70^. The most divergent region is the N-terminal part of the protein (region 1.1) involved in formation of transcription initiating complex and the spacer region [52], [53]. In spite of these differences, the *E. coli* RNAP can bind and transcribe *H. pylori* promoters both *in vivo* and *in vitro*, but not the other way around [52], [53].

### The T-tract modulates the RNAP α-subunits interaction to P*sabA*

**Figure 5:**
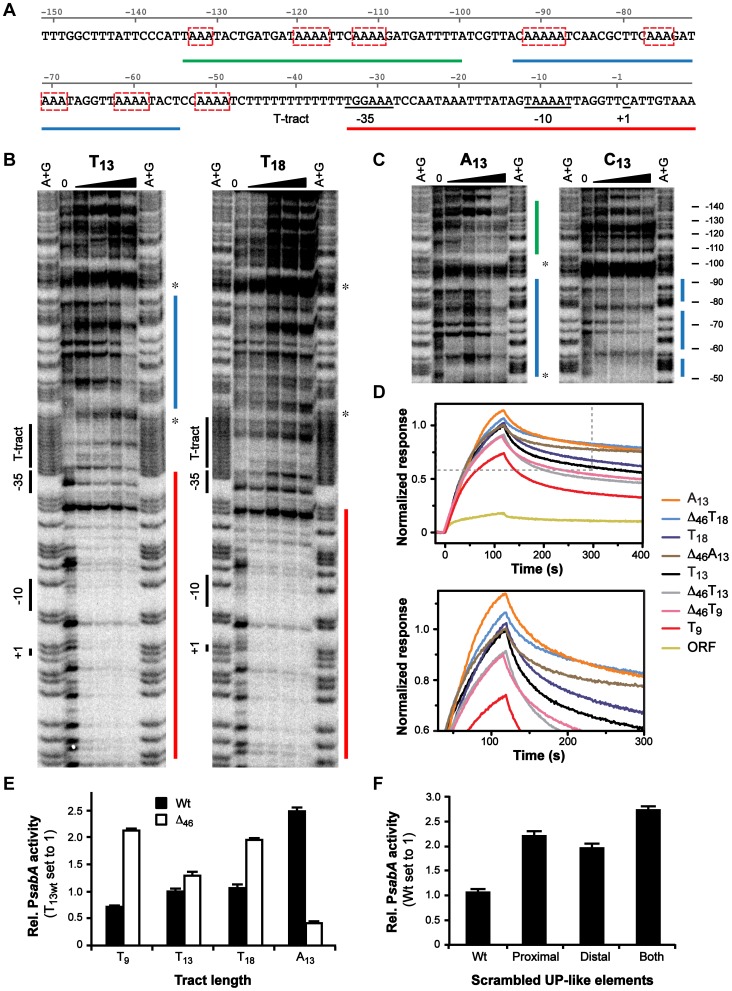
α-subunits of RNAP bind to A-boxes upstream of the T-tract. A) DNA sequence of the P*sabA* upstream region showing the predicted UP-like elements and multiple A-boxes (red boxes). Red, blue and green lines mark the interaction sites of σ^70^-RNAP found by Footprint analysis, correspondingly, see Fig. 5B–C. B–C) Mapping of the binding site for σ^70^-RNAP to P*sabA* DNA using DNase I footprint assay. 10 nM of [γ^32^P]ATP-labeled P*sabA* DNA (−166 to +74) were mixed with increasing concentrations of σ^70^-RNAP (0, 6.25, 12.5, 25, or 50 nM). The regions protected from DNase I cleavage are marked by red (core promoter), blue (proximal UP-like element) and green (distal UP-like element) lines. The positions of the T-tract, predicted −35 and −10, and +1 transcriptional start site, are indicated to the left. The stars mark the region of the promoter that was deleted in Δ_46_ variants (−97 to −49, see also Fig. S2 and S6A). Nucleotide positions, relative to the transcriptional start site, are shown to the right. D) Binding of σ^70^-RNAP (55 nM) to P*sabA* DNA (−166 to +74), with different repeat tract compositions and promoter mutant variants, analyzed by SPR. The sensorgrams show values normalized to that of the full-length T_13_-variant. Binding to a *sabA* CDS-fragment, also used in Fig. 4, is shown as a background curve in the top diagram. The bottom diagram is an enlargement of the dotted-lined square in the top diagram. E) Promoter activity of P*sabA*::*lacZ* transcriptional fusion plasmids, containing P*sabA* with proximal UP-like element deleted. The constructs contain different tract lengths and compositions (see Fig. 5B–C and S6A). Black bars represent wt promoters and white bars Δ_46_ variants, respectively. β-galactosidase assays were performed in the *E. coli* strain AAG1, with cultures grown to OD_600_ of 2 and analyzed as described in Materials and Methods. Data is presented as relative values with activity of P*sabA* T_13_ wt set to 1. F) Promoter activity of P*sabA*::*lacZ* transcriptional fusion plasmids, containing *sabA* promoter with scrambled UP-like elements. β-galactosidase assays were performed as described in Fig. 5E and data is presented as relative values with activity of P*sabA* wt set to 1.

Besides docking of the σ-factor to the core promoter (−35/−10 elements), RNAP binding can also include interaction of the C-terminal domain of the α subunits (αCTDs) to UP-elements located upstream of the core promoter [57]–[59]. To elucidate if the T-tract affects RNAP binding by influencing σ-factor or αCTDs binding, we obtained DNase I footprints of σ^70^-RNAP and P*sabA* DNA fragments with different repeat tract length and composition (T_9_, T_13_, T_18_, A_13_ and C_13_). The results showed strong binding to the core promoter (−35 to +20) with all variants, and no direct interaction to the T-, A- or C-repeat tracts (Fig. 5B, data not shown). Previous studies show that UP-elements positioned close to the −35 element have a larger impact on αCTDs binding than UP-elements located further upstream. UP-elements positioned upstream of −60 have not been shown to influence transcription of promoters in *E. coli* [59]. Interestingly, we could observe a clear DNase I protected region, positioned at −95 to −50, upstream of the repetitive tract (Fig. 5B–C, blue line) in some of the variants: T_13_, A_13_ and C_13_.

As the RNAP αCTDs often interact with AT-rich DNA sequences, we scrutinized the P*sabA* nucleotide content and it showed more than 74% A/T-residues. This can be compared to 61% A/T-content in the whole genome of *H. pylori* strains. Dissecting the P*sabA* region revealed two regions, upstream of the T-tract, with three to four short repetitive A-boxes in each region (Fig. 5A and S2, red boxes). This suggested that the RNAP αCTDs might interact to more than one site. Corroborating this, we could clearly observe an additional protected region in the A_13_ variant, located further upstream of the proximal region, positioned approximately at position −130 to −105 (Fig. 5C, green line). The footprint analysis revealed that several of these A-boxes were located within the DNase I protected regions observed in Fig. 5B–C. Two of A-boxes were perfectly phased, *i.e.* spaced by 10 bp, similar to an *E. coli* UP-element (NNAAAWWTWTTTTNNAAANNN). However, the regions containing the A-boxes are located further away from the core promoter and did not display complete sequence consensus to an UP-element. Therefore, we considered the regions containing the A-boxes in P*sabA* to be UP-like elements (Fig, 5A and S2, blue and green lines).

Based on these findings, we generated P*sabA* DNA fragments lacking the proximal UP-like element (Δ_46_ between −97 to −49), effectively positioning the distal UP-like element closer to the T-tract and the core promoter (Fig. 5A, green line). We speculated that the tract length/composition would optimize the RNAP αCTDs binding to the distal UP-like element differently in the Δ_46_ fragments, relative to the full-length fragments. As hypothesized, DNase I footprint analysis of the P*sabA* Δ_46_ fragments showed a stronger and more distinct protected region upstream of the repetitive tract as compared to the wt fragments, respectively (Fig. S6A). This was the case in all variants except for the Δ_46_T_9_ fragment. This further demonstrates the RNAP αCTDs capability to also interact with the more distal region. We also assayed the σ^70^-RNAP binding in an additional SPR-system where the same series of P*sabA* Δ_46_ fragments as above, were analyzed simultaneously as their respective wt fragments. We found that the σ^70^-RNAP bound stronger to the P*sabA* Δ_46_T_9_ DNA when the proximal UP-like element was removed, although we could not detect any interaction with the UP-like element (compare Fig. 5D and S6A). We also observed that RNAP bound slightly stronger to P*sabA* Δ_46_T_18_, corroborating the footprint results (Fig. 5D and S6A). Furthermore, the RNAP bound weaker to P*sabA* Δ_46_T_13_ and P*sabA* Δ_46_A_13_ (Fig. 5D), respectively, although we still could observe an interaction between RNAP to distal UP-like element. Next we analyzed promoter activity of these fragments lacking the proximal UP-like element as transcriptional *lacZ* fusions in *E. coli* and found that the expression patterns to large extent match the SPR results (Fig. 5E). We observed increased promoter activity from P*sabA* Δ_46_T_9_ and P*sabA* Δ_46_T_18_ variants, following the increased binding of RNAP, and we also found a concomitant decrease of P*sabA* Δ_46_A_13_ promoter activity. If the binding of the RNAP αCTDs were restricted to the proximal UP-like element, just upstream of the core promoter, the sensorgrams and promoter activities of all deletion fragments should have converged to identical ones, due to the interaction of σ^70^-subunit alone. Instead, the Δ_46_ fragments, with varying tract length/composition, still displayed different RNAP binding efficiencies and promoter activities, suggesting that the RNAP can bind to additional regions upstream of T-tract, corroborating the footprint results.

To confirm interaction of RNAP with the UP-like elements we scrambled the A-boxes by exchanging every second A in the A-box with a C or a G (see Materials and Methods for details). Promoter activity of P*sabA* DNA with scrambled UP-like elements (A-boxes) was analyzed as transcriptional *lacZ* fusions in *E. coli*. First, the four A-boxes in proximal UP-like element (Fig. 5A, blue line) or the three A-boxes in the distal UP-like element (Fig. 5A, green line) were scrambled separately. The results showed a clear effect on promoter activity, as expression from P*sabA* was increased in both variants by 2-fold (Fig. 5F). Concurrently, when both UP-like elements were changed an even more pronounced up-regulation (3-fold) of P*sabA* activity was observed. Regulation of transcriptional output from a promoter is a complex multi-step process, involving binding of RNAP, separation of DNA strands, initiation of transcription and promoter escape. Ellinger *et al* showed that depending on a promoter's rate-limiting step in the pathway to productive transcription, A-tracts/UP-elements can either activate or inhibit promoter activity [60]. To determine at what step P*sabA* activity is limiting, extensive *in vitro* transcriptional studies are required.

Taken together, our results show that the two UP-like elements are important for P*sabA* activity and that the RNAP interacts directly with both regions upstream of the core promoter. The T-tract length modulates this interaction, by changing the affinity of the RNAP to the promoter, and thereby affecting P*sabA* activity. During transcription initiation the DNA is wrapped >300° around the RNAP [61] and although most of the effect of UP-like elements occurs via interaction with the αCTDs, we cannot exclude the possibility that some of the upstream sequences is also interacting with other surfaces on the RNAP.

### The T-tract length influences local DNA structure

**Figure 6:**
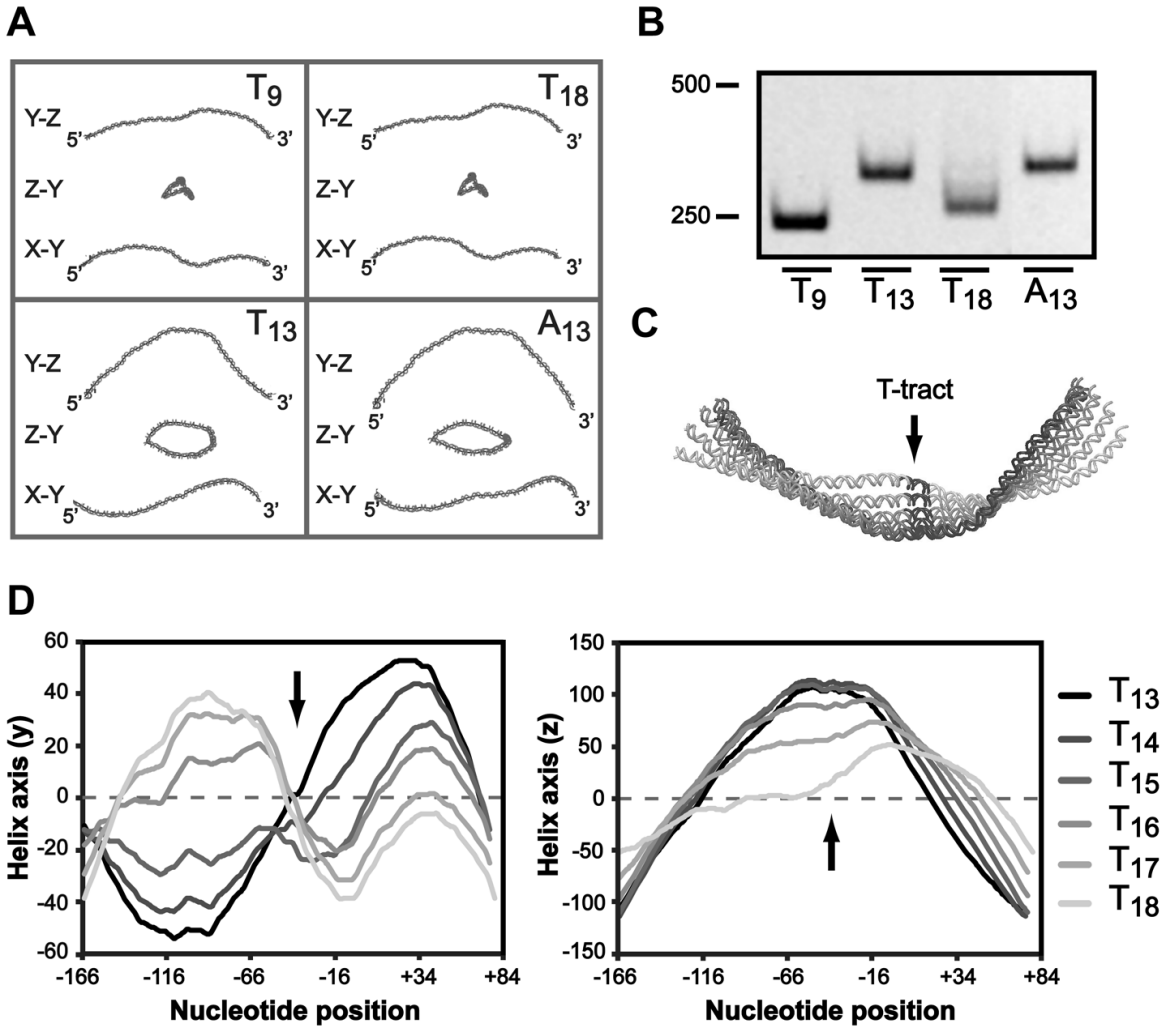
The T-tract length affects the local DNA structure of the *sabA* promoter. A) *In silico* DNA structure predictions of the P*sabA* (−166 to +74) harboring different repeat tract lengths and nucleotide compositions, using the AA Wedge model (http://www.lfd.uci.edu/~gohlke/dnacurve/). The analyzed DNA fragments contain T_9_-, T_13_-, T_18_- or A_13_-tracts. The structures shown represent the 3D DNA helix backbone, displayed in three dimensions. B) Gel migration of DNA fragments containing the P*sabA* with different repeat tract lengths and compositions. The DNA samples, same set as in Fig. 6A, were run at 4°C in a Tris-Glycine 4.5% polyacrylamide gel that was stained with GelRed. The DNA size marker (bp) is shown to the left. C) Alignment of P*sabA* DNA fragments analyzed as pdb structures in the Protean 3D software (Lasergene, DNASTAR). The T-tract was extended by 1 thymine (T) at a time (from 13 to 18), and predictions were made as in Fig. 6A. The image shows one view from a selected angle, with the T-tract marked in black and by an arrow. The different T-variants are labeled in shades of gray, see Fig. 6D. D) A 1D plot of the shape of the P*sabA* DNA helix, visualized in the y orientation (left diagram) and in the z orientation (right diagram). The coordinates were generated from the predictions in Fig. 6C. The black arrows mark the position of the T-tract in the DNA helix.

Short phased A-tracts act as major determinants of DNA curvature by forming intrinsically bent DNA and depending on the tract periodicity this alters the DNA structure to different conformations [62]–[64]. Our results suggest that the A-boxes in the UP-like elements upstream of the T-tract is important for P*sabA* activity, therefore we hypothesized that the effect on RNAP binding can be a result of changes in local DNA structure. We made *in silico* structure predictions of the P*sabA* DNA (−166 to +74) of different T-variants (T_9_, T_13_, T_18_ and A_13_), which supported this hypothesis by showing structural changes of the DNA in several orientations (Fig. 6A). This was further supported by PAGE separation of P*sabA* DNA fragments with varying tract length/composition where we observed altered migration patterns that can be explained by structural differences rather than by the small differences in size (Fig. 6B). The T_9_ and T_18_ variants have an apparent similar structure (Fig. 6A), although the *sabA* expression are slightly different, low vs. intermediate (Fig. 2). Possibly, the longer T-tract in T_18_ may give a more flexible DNA that allows for some contact between the RNAP αCTDs and the UP-like elements, as our SPR and footprint data from the wt and Δ_46_ P*sabA* DNA suggested (Fig. 5D and S6A). To further look into this, we made *in silico* structure predictions of P*sabA* DNA with sequential nucleotide extensions in T-tract length (T_13_ to T_18_). Evidently, a distinct 3D DNA structure was observed for each variant (Fig. 6C), since the DNA was converted both in the y and in the z orientation by each thymine addition (Fig. 6D). This is in line with the alterations in mRNA levels we detected in the *H. pylori* T-variants with one deleted (T_12_, 60%) or two added (T_15_, 75%) T's, as compared to the wt (T_13_, 100%, Fig. 2C). This illustrates the influence of small alterations in T-tract length on the final SabA expression and sLex binding activity in *H. pylori*. This is also visible in the heterogeneous populations isolated post-infection (Fig. 3).

Some of the A-boxes, described in the preceding section, have a perfectly phased location (10–11 nucleotides in between) in the DNA helix (Fig. 5A and S2). We propose that the A-boxes are interaction sites for RNAP αCTDs and also contribute to the intrinsic DNA curvature in the promoter (Fig. 5–6). Such curvature has previously been shown to affect both binding of RNAP (formation of closed complex), melting of DNA strands (formation of open complex), release from promoters (promoter escape) and binding of trans-acting factors, which argues that upstream static DNA bends can influence promoter activity at several levels [65]. Structural predictions of P*sabA* fragments lacking the proximal UP-like element showed major structural alterations in P*sabA* DNA as compared to the wt fragments (Fig. S6B). This explains the SPR and promoter activity results where we observe a stronger interaction and increased promoter activity with low-expressing T_9_ and T_18_ variants as this region is missing (Fig. 5D–E). Probably, the A-boxes in the distal UP-like element is in a more favorably phasing in the Δ_46_T_9_ and Δ_46_T_18_, promoting DNA curvature and optimal contact to RNAP, than in the Δ_46_A_13_ variant (Fig. 5E and S6B). The overall effect on promoter activity observed in these variants is probably due to a combination of the changed RNAP binding and DNA structure. Structure predictions of the scrambled UP-like elements revealed that it is the A-box located between the T-tract and the proximal UP-like element that has most impact on DNA structure (Fig. S6C). This A-box is missing in the Δ_46_ fragments, probably resulting in observed changes in DNA structure (Fig. S6B) but was kept unchanged in our scrambled UP-like elements (Fig. S6C). In conclusion, our results suggest that the T-tract length drives the A-boxes into different phasing of the DNA, thereby altering the three-dimensional architecture of P*sabA* DNA. Furthermore, this changes the angular orientation between the core promoter and UP-like elements resulting in enhanced or decreased interaction of RNAP with DNA, giving the observed multiphasic expression pattern of SabA protein and sLex-receptor binding activity (Fig. 2).

### The T-tract length affects P*sabA* activity without involvement of known DNA binding proteins

Not only AT-rich DNA is known to bend DNA, but also binding of nucleoid-associated proteins (NAPs). SSRs positioned upstream of −35 promoter elements frequently influence the binding of a trans-acting regulatory factor exemplified by; the TAAA tract of the *nadA* promoter in *Neisseria meningitidis*, affecting binding of integration host factor (IHF); the GAA tract of p*MGA* in *Mycobacterium gallisepticum*, affecting binding of a putative regulator HAP; and the A-tract of P*atzDEF* in *Pseudomonas putida*, affecting binding of AztR [48], [66], [67]. Typical for many of the classical trans-acting transcriptional regulators in other species, such as H-NS, cAMP receptor protein CRP, and LysR-type regulators, are their ability to interact with AT-rich DNA [68]–[70]. Though, there is no H-NS or IHF homolog present in *H. pylori*, two other NAPs have been described; the HU homolog Hup [71], [72] and the Dps homolog NapA [73], [74]. HU is one of the NAPs conserved in eubacteria.

In order to explore if these DNA binding proteins affect *sabA* expression, we constructed *hup* and *napA* mutants in strain SMI109 and analyzed changes in expression by RT-qPCR (mRNA levels), Western (protein expression) and RIA (receptor binding activity). However, we could not observe an effect on *sabA* expression, at any level, in either the *hup* or the *napA* mutant (Fig. S7B–C). We also analyzed *sabA* expression in *hup* mutants with various T-tract lengths, and again no effect was observed (Fig. S7D–E). We cannot yet exclude that no additional factors are involved in regulating *sabA* mRNA levels in combination with the T-tract. To our knowledge the only trans-acting factor that affects SabA expression is the acid responsive ArsRS system that represses SabA expression at acidic conditions [33]. How this repression operates in molecular terms and if the regulation occurs by direct interaction with P*sabA*, is not yet known. Nonetheless, our results show that the T-tract length located adjacent to the −35 element of the *sabA* promoter affects binding of the RNAP and thereby the transcriptional output, without involvement of any known DNA binding proteins.

The recurrent multiphasic SabA expression pattern observed in the T-variants supports the hypothesis that it is the structure of promoter DNA and RNAP interaction, rather than binding of a trans-acting factor, that is important for expression. The multiphasic pattern was much more pronounced in *H. pylori* (Fig. 2) than when promoter activity was analyzed in *E. coli* (Fig. S1C). Two of the T-variants, T_18_ and C_13_, displayed divergent expression levels in *H. pylori* as compared to the *in vitro* data (compare Fig. 2 and 4). Nonetheless, SPR analysis of σ^70^-RNAP binding and the promoter activities analyzed in *E. coli* show comparable results (Fig. 4A–B). It is therefore tempting to speculate that the dissimilarities could be due to structural differences of the *E. coli* and *H. pylori* RNAPs. Our results indicate that it is the interaction between α-subunit of the RNAP and the UP-like elements that is affected by the T-tract length, through change in DNA structure. Homology predictions has shown that the RNAP α- and ω-subunits are more divergent between different bacterial species than the remaining subunits [75] and thus, interaction of RNAP to DNA structures or DNA binding trans-acting factors might deviate from *E. coli*. Borin *et al* showed that the linker region between the αCTD and αNTD is longer in *H. pylori* compared to the *E. coli* α-subunit. The *H. pylori* αCTDs have an additional amphipathic helix in the C-terminal [76], which could explain why the highest expression in *H. pylori* is the T_13_ whereas it is T_18_ in *E. coli*. The *H. pylori* α-subunit should, due to these structural differences, be able to reach further upstream than the *E. coli* one, to make contact with the UP-like elements or potential trans-acting factors.

### T- or A-tracts adjacent to −35 elements affect transcription in *H. pylori*

SSR motifs located between the −35 and −10 promoter elements affect docking of the RNAP σ-factor, motifs located upstream of the −35 element affect binding of regulatory factors [6], and as we show here for *sabA*, motifs located adjacent to the −35 element adjust transcription initiation by affecting local DNA structure. To dissect if this finding is a general phenomenon in *H. pylori*, we searched the genome of strain 26695 for additional genes with T- or A-tracts (>9 nucleotides) close to the −35 element. Among the predicted promoters of *H. pylori* [77], we found twenty-five genes with appropriately located T- or A-tracts (Table 2). Interestingly, loci encoding outer membrane proteins were again overrepresented among these genes (15 of 25 genes).

**Table 2:** Loci in strain 26695 with T- or A-tracts (≥9 bp) located close to −35 promoter elements.

Gene no	Gene	No. T's/A's	Position	HP no	Gene	No. T's/A's	Position
HP_0009	*hopZ*	14A	−35/−10	HP_0811	hyp	14A	≪−35
HP_0025	*hopD*	15T	<−35	HP_0812	HPnc4160	14T	−35/−10
HP_0209	*hofA*	11T	<−35	HP_0876	*frpB*	16T	−10>
HP_0227	*hopM*	14T	<−35	HP_0896	*babB*	14A	−35/−10
HP_0228	hyp	14A	≪−35	HP_0912	*alpA*	13T	≪−35
HP_0229	*hopA*	9A	−35	HP_0914	*hopG*	13T	−35/−10
HP_0317	*hopU*	9A	−35/−10	HP_1105	LPS	15T	−35/−10
HP_0349	*pyrG*	15T	≪−35	HP_1106	hyp	15A	≪−35
HP_0350	hyp	15A	<−35	HP_1206	thr tRNA	10T	−35/−10
HP_0547	*cagA*	14A	−35	HP_1243	*babA*	9A	−35/−10
HP_0722	*sabB*	16T	<−35	HP_1342	*hopN*	14A	≪−35
HP_0725	*sabA*	14T	<−35	HP_1400	*fecA*	16A	−10>
HP_0733	hyp	13T	−35/−10				

Position abbreviation; −35/−10: between elements, <−35: ≤20 nucleotides upstream of −35, ≪−35: >20 nucleotides upstream of −35, −35: overlapping with −35, −10>: downstream of −10.

Among the twenty-five loci, nine had a T- or A-tract located between the −35 and −10 elements, two replaced the −35 element, six were located approximately 30, 31, 59, 68 and 86 nt upstream of −35 element, respectively, and two were located downstream of the transcriptional start site. Furthermore, five loci had T- or A-tracts located adjacent (<20 nt) to the −35 element, similar to that of *sabA*: *sabB* (HP_0722), *hopD* (HP_0025), *hofA* (HP_0209), *hopM* (HP_0227), and *hp_0350* (Table 2). We compared the tract lengths of these five loci in the forty-nine publically available genome sequences (Table S1). Our comparison showed that all T- or A-tracts displayed great length variability, in line with the individual selection and stochastic switching hypotheses discussed in preceding sections (Fig. S8).

**Figure 7:**
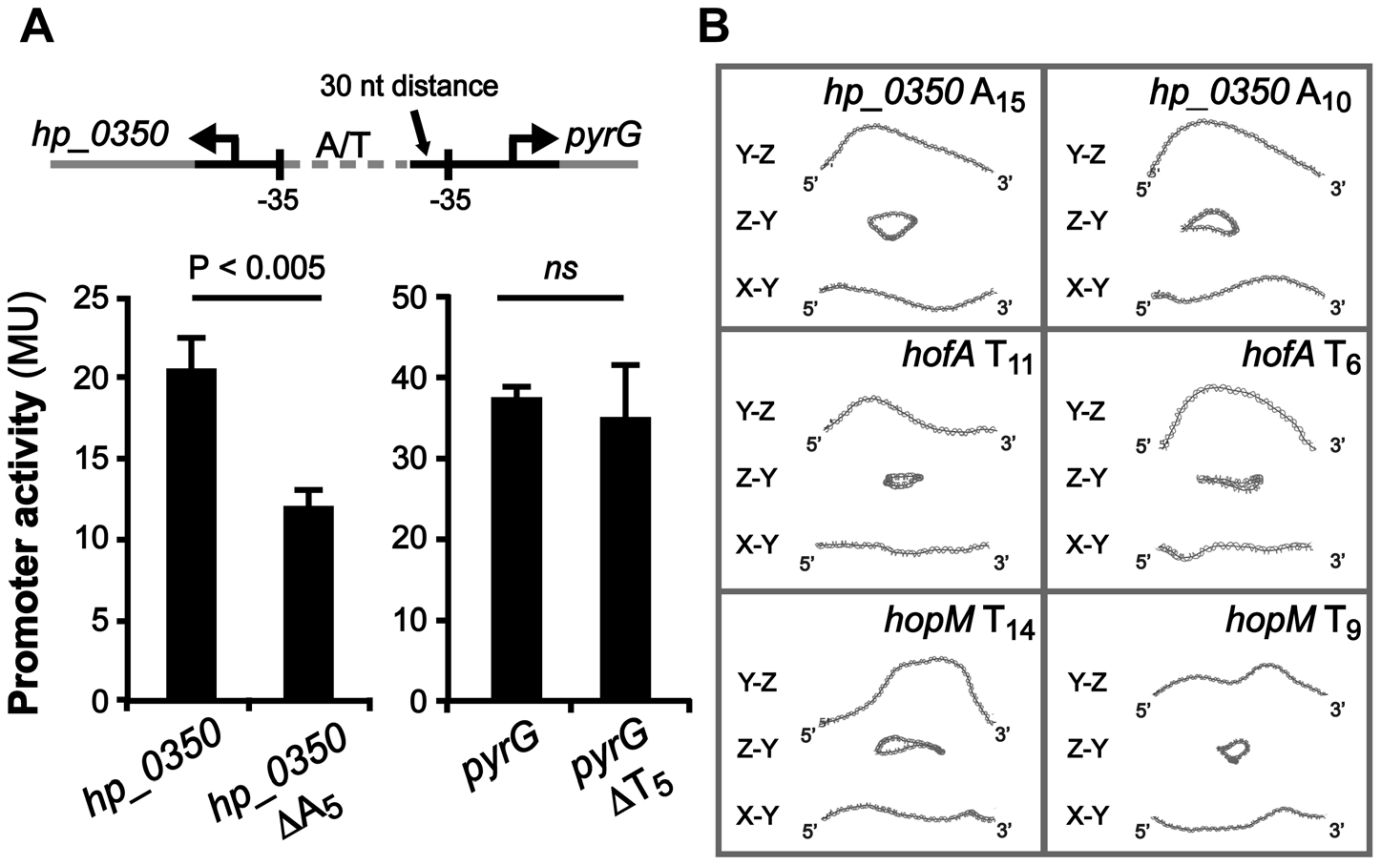
*hp_0350* promoter activity is affected by the A-tract located adjacent to the −35 promoter element. A) Effects on *hp_0350* and *pyrG* promoter activities by the length of the repeat tract located in their divergent promoter regions (A_14_/T_14_ [wt] vs. A_9_/T_9_ [Δ_5_]). Strains were grown in Brucella broth at 37°C in 24-well plates under microaerophilic conditions Expression from the *hp_0350*::*lacZ* and *pyrG*::*lacZ* reporters in strain SMI109 shown are from samples collected in logarithmic growth phase (OD_600_ of 0.2, Fig. S7A). Illustration shows the position of the repeat tract, relative to the −35 elements, of each gene. DNA sequence alignment of the *hp_0350*/*pyrG* promoter regions from 45 different strains is shown in Fig. S9. B) *In silico* DNA structure predictions of the *hp_0350*, *hofA* (HP_0209) and *hopM* (HP_0227) promoter regions based on sequences from strain 26695. The analysis were performed as in Fig. 6A. Images in the left panel show DNA structures with wt tract lengths, and in the right panel, the structures of promoter DNA with 5 nucleotide shorter repeat tracts.

We also found four intergenic regions with predicted divergent transcriptional start sites, and T- or A-tracts located in between their respective promoters (Table 2). The adjacent genes, *hp_0350* (unknown) and *pyrG* (CTP synthase), have an A-tract located just three nucleotides upstream of the −35 element of *hp_0350*, and a T-tract positioned 30 nucleotides upstream of the −35 element of *pyrG* (Fig. 7A). These two loci were found in all sequenced *H. pylori* genomes, and additionally, in other *Helicobacter* species such as *H. acinonychis* isolated from cheetahs and *H. cetorum* isolated from Atlantic white-sided dolphins (Table S1). In order to test if the length of the T- or A-tract influences the expression of these genes, we created *lacZ* transcriptional fusion plasmids of *hp_0350* and *pyrG* promoter regions from strain SMI109 (A_14_/T_14_). Additionally, we constructed the corresponding promoter variants with 5 nucleotides shorter A- or T-tracts (A_9_/T_9_), *i.e.* half the distance between min/max expression levels observed for *sabA* (Fig. 2). The promoter activities were analyzed by β-galactosidase assay in *H. pylori*, and as hypothesized the *hp_0350* promoter showed distinct differences in activity in the A-tract length variants, whereas in contrast, the *pyrG* promoter activity remained unaffected by the change in T-tract length (Fig. 7A). The experiment was repeated at different growth phases and these *lacZ* fusions were also analyzed in *E. coli*, with the same results (data not shown).

In addition, we made *in silico* DNA curvature predictions of three additional promoter regions (*hp_0350*, *hofA* and *hopM*) and found that the DNA structure was affected as the length of the repetitive tract was decreased by 5 nucleotides (Fig. 7B), just as we observed for *sabA* (Fig. 6A). Alignment of the *hp_0350* promoter sequences also revealed high homology between different strains (Fig. S9) and we could observe conserved A-boxes located upstream of the A-tract, forming potential UP-like elements, where the RNAP α-subunits likely interact. Our findings suggest that poly A- or T-tracts located adjacent to −35 elements fine-tune promoter activity, and thereby mRNA levels, by changing the DNA structure as the tract length is altered.

### Concluding remarks

**Figure 8:**
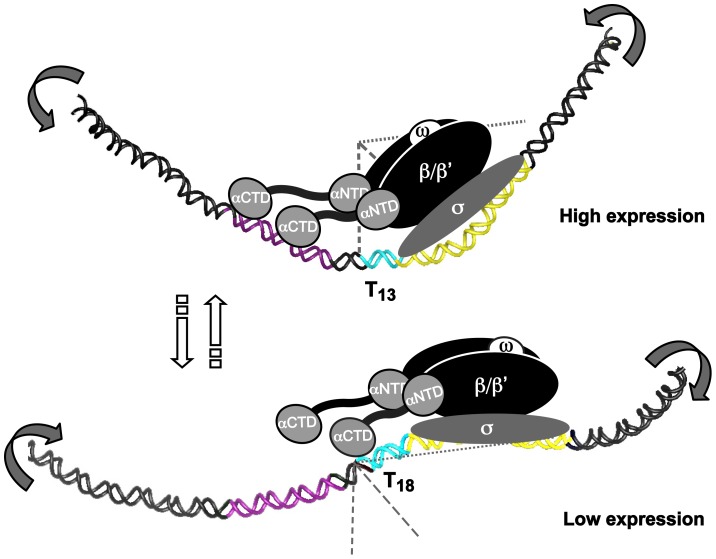
T- or A-tracts, adjacent to −35 elements, regulate gene expression by a rheostat-like mechanism. Schematic overview of the T-tract rheostat using the *sabA* promoter as a model. The predicted interaction of the RNA polymerase with *sabA* promoter, harboring different T-tract lengths and thereby different local DNA structure, is depicted in the model. The illustration shows the high-expressing T_13_-variant and the low-expressing T_18_-variant. The region containing the A-boxes, *i.e.* the proximal UP-like element, is marked in purple (−90 to −50), T-tract in blue, and the core promoter (−35 to +20) in yellow. Bent arrows indicate the change in local DNA structure that occurs in two orientations as the T-tract length is altered. This is a variable process as the T-tract length can both be lengthened and shortened, as a result of slipped strand mispairing during replication.

In conclusion, we describe a general mechanism where certain simple sequence repeats (SSRs) in *H. pylori* changes the local DNA structure, which by a rheostat-like mechanism affects interaction of the RNAP, to fine-tune gene expression via slipped strand mispairing (SSM). For SabA, optimal T-tract length (T_13_ in strain SMI109) positions the upstream-located A-boxes in a favorable phasing, aligning the UP-like elements and the core promoter, to enhance RNAP interaction, and resulting in higher promoter activity (Fig. 8). Thus, low transcriptional activity occurs when the UP-like elements and the core promoter are skewed relative each other, and the interaction of the RNAP α-subunits to the UP-like elements is disturbed (Fig. 8). Each T-tract length variant displayed a unique 3D DNA structure, contributing to the multiphasic transcriptional output observed from the *sabA* promoter. The mechanism described in this paper is possibly of significance to other bacterial systems that like *H. pylori* have a limited repertoire of trans-acting transcription factors and numerous SSRs, *e.g. Haemophilus influenzae* and *N. meningitidis*.

SabA is just one, of many outer membrane proteins in *H. pylori*, which have SSRs in its inter- and intragenic sequences. Intragenic CT-tracts have been reported for loci encoding BabA, BabB, SabB, OipA and HopZ, and we show that a set of them also harbor T- or A-tracts in their promoter regions, respectively. Taken together, all the SSR regions found in a genome create multiple contingency loci of hypermutable DNA that via different mechanisms blindly mediate causal and heritable genotypes, and contribute to stochastic switching. This cost-beneficial approach meets the need to control gene expression at various levels and can despite the lack of specific trans-acting regulators pilot persistent infections in fluctuating host environments through production of heterogeneous bacterial populations of best-fit phenotypes.

## Materials and Methods

### Ethical statement

The animal studies were approved by the Animal Care and Use Committee of Umeå University and by the ethical committee of Swedish Board of Agriculture (Decision No. A120-06). Experiments were conducted in accordance with Guidelines for Care and Use of Laboratory Animals.

### Growth conditions and strains

Bacterial strains used in this study are listed in Table 1. *H. pylori* strains were routinely grown on Brucella agar (Difco) supplemented with 10% citrated bovine blood (Svenska Labfab), 1% IsoVitox (Becton Dickinson, US) and an antibiotic mix (4 mg/L amphotericin B, 5 mg/L trimethoprim and 10 mg/L vancomycin). When needed, *H. pylori* strains were grown in culture medium containing Brucella Broth (Difco), 1% Isovitox and 10% fetal calf serum (Gibco). Plates or broth were, when required, supplemented with chloramphenicol (20 mg/L) and/or kanamycin (25 mg/L). Bacteria were grown at 37°C under microaerophilic conditions (5% O_2_, 10% CO_2_, and 85% N_2_). For the analysis of *sabA* mRNA levels, protein expression and sLex-receptor binding, equal amounts of each strain were re-plated onto Brucella blood agar plates, and the bacteria were collected after 16 h of growth, in order to have all strains in the same growth phase. For the Δ*hup* strain the plates were left for 40 h due to the delayed onset of growth (Fig. S7A). *E. coli* strains were cultured in Luria broth (LB) agar at 37°C, supplemented with carbenicillin (100 mg/L) and/or kanamycin (25 mg/L). Growth was measured by OD at 600 nm using the spectrophotometer Ultrospec2100 PRO (GE healthcare).

SMI109 Δ*sabA* was created by transformation of a plasmid containing the Δ*sabA*::*cam* construct [13]. Deletion of the *sabA* gene, loss of sLex-receptor binding, and absence of SabA expression was verified by PCR, RadioImmunoAssay (RIA), and immunoblot assays, respectively. We also determined, by diagnostic PCR, as previously described [18], that the *sabA* homolog *sabB* is absent in strain SMI109. SMI109 Δ*hup* was created by transformation of a Δ*hup*::*kan* PCR fragment generated by *hup-1* and *hup-5* primers, and pAAG178 as template. SMI109 Δ*napA* was created by transformation of a Δ*napA*::*kan* PCR fragment generated by *napA1F* and *napA1R* primers, and pBlue-Δ*napA*::*kan* [78] as template. Deletion of the *hup* and *napA* genes was verified by PCR using *hup-2*/*hup-in* and *napA2F*/*napA2R* primers, respectively. Plasmids used are shown in Table 1 and primers in Table S2.

J99^StrR^ was constructed by transformation of plasmid pEG21 (a kind gift from Prof Rainer Haas, Ludwig Maximilians University, Munich, Germany) into J99. The bacteria were plated on plates containing 500 mg/L streptomycin to obtain single colonies and a *sabA* T_17_ and CT_8_-off clone was selected and used for animal studies.

SMI109 *pyrG*::*lacZ* and *hp_0350*::*lacZ* strains were constructed by transformation of pAAG202-205 plasmids into SMI109. Correct incorporation in the chromosome was verified by PCR.

### Genetic techniques

Basic molecular genetic manipulations were performed essentially as described previously [79]. Genomic DNA was isolated as previously described [80] from bacteria grown on plate. Polymerase chain reactions (PCR) were carried out according to the manufacturer's instruction, using GoTaq polymerase (Promega) or Phusion Hot start DNA polymerase (Thermo Scientific), on a MJ PTC-200 thermal cycler (MJ Research). For isolation of plasmid DNA, the E.Z.N.A Mini and Midi column plasmid purification kits were used and purification of PCR products were done with the E.Z.N.A Cycle Pure or Gel Extraction kits (OMEGA *bio-tek*, USA). Plasmids and/or PCR products were sequenced at Eurofins MWG Biotech (Germany).

### Construction of *lacZ* transcriptional fusion plasmids

The *sabA* transcriptional *lacZ* fusion plasmids were obtained by cloning a PCR-amplified fragment (*sabA-1* and *sabA-3*) spanning 310 bp of the *sabA* promoter region and 8 bp of the CDS (−244 to +74) between the *EcoR*I-*BamH*I sites in pRZ5202 creating a transcriptional fusion (Fig. S1A). As template, genomic DNA from different *H. pylori* strains (26695, J99, G27, 17875/sLex and SMI109) were used. Site-directed mutagenesis, using primers spanning ∼20 bp on either side of the T-tract (see example *sabA*-Tf/*sabA*-Tr in Table S2), were used to change the length of the T-tract in the *sabA*::*lacZ* promoter fusions.

The Δ_46_ promoter fragments were constructed with over-lapping PCR using primers P163–165 and P167 (different variants), and 162 (Table S2). As template P*sabA* DNA from SMI109 was used. Mutations were verified by sequencing and a PCR-amplified fragment (*sabA-1* and *sabA-3*) was cloned between *EcoR*I-*BamH*I sites in pRZ5202, creating *lacZ* transcriptional fusions. For SPR and footprint analysis, PCR fragments generated with primers *sabA-5* and *sabA-8* were used.

Scrambling of the A-boxes in UP-like elements of P*sabA* was generated by site-directed mutagenesis, using primers spanning the proximal (Amut1) or/and distal (Amut2) elements (Table S2). As template P*sabA* DNA from SMI109 cloned in pUC19 was used. Mutations were verified by sequencing and a PCR-amplified fragment (*sabA-1* and *sabA-3*) was cloned between *EcoR*I-*BamH*I sites in pRZ5202, creating *lacZ* transcriptional fusions.

The *hp_0350* and *pyrG* promoter *lacZ* fusion plasmids were obtained by cloning the PCR-amplified fragments (*hyp F*/*hyp R* or *pyrGp F*/*pyrGp R*) spanning the *hp_0350/pyrG* promoter region between *Sal*I-*Bgl*II sites in pBW. As template, genomic DNA from strain SMI109 was used. Stitch PCR using primers spanning ∼20 bp on either side of the T- or A-tract (*pyrG* 9Tf/*pyrG* 9Tr), were used to change the length of the T- or A- tract in the *pyrG*::*lacZ* and *hp_0350*::*lacZ* promoter fusions.

### Construction of T-tract mutants in *H. pylori*

Isogenic *sabA* repeat tract variants were constructed by contraselection in strain SMI109, as previously described [81]. In short, the *sabA* promoter region was removed and replaced by an antibiotic resistance cassette, generating the SMI109Δ*sabA*::*rpsLCAT* strain, using primers LA-F, LA-R, RA-F, RA-R, *rpsLCAT*-F, *rpsLCAT*-R. PCR fragments harboring the *sabA* promoter region, with different lengths or composition of the repeat tract, were generated by stitch PCR using (P93, Tf, Tr, P96) and transformed into the SMI109Δ*sabA*::*rpsLCAT* strain, to replace the antibiotic cassette. Tf and Tr refer to the complementary primers spanning the region determining the tract length or composition (see Table S2). Clones were verified by PCR and sequencing using P93 and P96 primers. The protocol was optimized to fit the SMI109 strain by first determining the frequency of false positive clones (as contraselection is somewhat leaky). The amount of bacteria that corresponded to <10 false positives (in mock transformation) was used for transformation, which greatly facilitated the yield of mutants.

### Construction of *hup* deletion/insertion fragment

A deletion/insertion fragment of the *hup* gene from SMI109 was generated by removal of the *hup* CDS and replacing that with a kanamycin resistance cassette. DNA regions upstream and downstream of the *hup* gene (HP_0835) were amplified by PCR using SMI109 genomic DNA as template, *hup-1*/*hup-3Km* and *hup-4Km*/*hup-5* primers generating PCR fragments with flanking regions homologous to a kanamycin cassette. The kanamycin cassette from pKD4 was amplified using *Km-up*/*Km-dn* primers. The three PCR fragments were stitched together using equimolar amounts of each PCR fragment and *hup-1*/*hup-5* primers. The *hup* deletion/insertion fragment was cloned in *Sma*I cut pUC19, generating the plasmid pAAG178, and analyzed by PCR and sequencing.

### RadioImmunoAssay (RIA)

Measurement of binding to soluble ^125^I-sLex-receptor conjugate was performed as previously described [82]. Samples were assayed in duplicates and minimum two independent sets of each experiment, plotted with standard deviations.

### Immunoblot analysis

Immunoblot analysis was performed as previously described [17]. Antibodies against SabA (AK278) and AlpB (AK262) [83] was used in combination with secondary α-rabbit IgG-HRP (DAKO A/S, Denmark). Blots were developed with SuperSignal (Pierce, Rockford, IL) ECL and detected on High Performance Chemiluminescence film (GE Healthcare). SabA protein densities were measured by ImageJ software (NIH) and normalized to the corresponding PAGE Blue stained SDS-PAGE gel (for Fig. 1A) or AlpB protein density (Fig. 2A, 4C, S6C and S6E) to calculate relative protein expression levels (Fig. S10).

### β-galactosidase assay

β-galactosidase activity measurements were performed as described by Miller [84]. Data shown are mean values of duplicate determinations of at least three independent experiments, plotted with standard deviations.

### RNA isolation

Total RNA was extracted using the SDS/hot phenol method, as previously described [85]. Contaminating DNA was removed by 10 U DNase I (Ambion 2 U/µl) treatment for 10 min at 37°C, followed by phenol/chloroform extraction. Quality and concentration of the total RNA was examined in a 1.2% agarose gel and by measurement on a micro-spectrophotometer (Nanodrop, ND-1000). The total RNA was stored at −80°C until used.

### 5′- Rapid Amplification of cDNA Ends (5′-RACE) and primer extension

The 5′-RACE analysis was made using FirstChoice RLM-RACE kit (Ambion) according to the manufacturer's protocol. In brief, ∼1 µg of total RNA isolated from different *H. pylori* strains was used in each ligation and cDNA synthesis reaction. For the first PCR reaction RACE-outer and AB35 primers were used, and for the second PCR reaction RACE-inner and J99-8 primers were used. The PCR fragment of expected size was cloned in *Sma*I cut pUC19 and sequenced using universal M13F/R primers.

Primer extension analysis was done as previously described [86] using total RNA samples from *H. pylori* and *E. coli* (20 µg), and [γ-^32^P]-ATP kinase-labeled *sabA-8* primer.

### cDNA synthesis and quantitative real-time PCR (qPCR) analysis

The cDNA synthesis and RT-qPCR analysis was performed in accordance to the MIQUE guidelines [87]. Total RNA was isolated from *H. pylori* strains grown on Brucella blood agar plates (as described above). Before cDNA synthesis the total RNA (250 µg/µl) was treated an extra time with Turbo DNase I (Ambion) to remove any residual DNA. cDNA synthesis was performed in 20 µl reactions using 500 ng Turbo DNase treated total RNA, Transcriptor First Strand cDNA Synthesis kit (Roche Applied Science) and random hexamers (60 µM) provided with the kit, according to the manufacturer's protocol. cDNA synthesis was performed at 25°C for 10 min and at 55°C for 30 min. The enzyme was inactivated at 85°C for 5 min. The cDNA was diluted with 80 µl DEPC MQ before it was used as template in qPCR reactions.

Quantitative real-time reverse transcriptase PCR (RT-qPCR) was used to determine the mRNA levels of *sabA* in different *H. pylori* strains, and the primers used are listed in Table S2. As reference genes, expression of *gyrA*, *ppk* and *rrnA* were simultaneously analyzed. RT-qPCR analysis was done in 20 µl reactions in 96-well plates using cDNA from 10 or 100 ng RNA as template, 2× FastStart Essential Green Master (Roche Applied Science), gene specific primers (5 µM each) and LightCycler 96 instrument (Roche Applied Science). Cycling conditions were; 10 min at 95°C and 40 cycles of; 20 s at 95°C, 20 s at 58°C and 20 s at 72°C. Fluorescence was detected at the end of each extension step, and the Cq values and relative ratios were calculated using the LightCycler 96 software (Roche Applied Science). After each run a melt curve analysis was performed and the size of the PCR products were analyzed by gel electrophoresis. In each run, a non-template control for each cDNA sample (NTC, RNA only), and a PCR negative control was included for each primer pair. At least duplicate samples were analyzed in each experiment and expression ratios were determined from at least two biological replicates. The PCR efficiencies were determined using standard curve analysis and cDNA from SMI109 as template, and were as follows: *sabA*-1 1.98±0.09, *sabA*-2 1.99±0.06, *ppk*-2 1.99±0.05, *gyrA*-1 1.96±0.03 and *rrnA*-2 1.90±0.03.

Semi-quantitive RT-PCR analysis was performed using the same cDNA and primers as in RT-qPCR analysis. 20 µl reactions, using cDNA from 100 ng of RNA as template, Phusion Hot Start DNA polymerase (Thermo Scientific) and gene specific primers (5 µM each), were run on MJ PTC-200 thermal cycler (MJ Research). Cycling conditions were; 30 s 98°C followed by 10 (*rrnA* amplicon) or 20 (*sabA* and *ppk* amplicons) cycles of 15 s at 98°C, 30 s at 55°C and 30 s at 72°C. 5 µl of each PCR reaction was separated in a 1.5% agarose TAE gel, stained with GelRed (Biotium) and scanned using the Kodak Image station 2000R.

### FITC-labeled bacteria overlaid on human paraffin tissue sections

Human gastric tissue was deparrafinized and incubated with blocking buffer (1× phosphate-buffered saline, 0.05% Tween-20 and 1% periodate-treated BSA) for 1 hour. Thereafter, FITC-labelled bacteria, prepared as described previously [12], was added and incubated for 3 hours. Tissue sections were washed in washing buffer (1× phosphate-buffered saline, 0.05% Tween-20), mounted with fluorescent mounting medium (DAKO), and analyzed for binding using Zeiss Axio Imager Z1 system and AxioVision software (Zeiss, Germany).

### Animal experiments and collection of bacterial sweeps

FVB/N male mice (6–8 weeks of age), transgenic for the human α-1,3/4-fucosyltransferase gene, resulting in the expression of ABO Lewis blood group antigen in the epithelial lining of the stomach, were used in this study [40]. Transgenicity was confirmed as described previously [40]. Mice were bred and kept in separate cages during the study and kept on a 12-h light-dark cycle. Water and standard pellet diet was provided *ad libitum*. Animals were infected with 2×10^8^ *H. pylori* (J99^StrR^ *sabA* T_17_ and CT_8_-off), twice a week during two weeks, via oro-gastrical gavage. To evaluate the *H. pylori* infection load, mice were sacrificed 4 weeks post-infection and stomach tissue samples were collected and quantitatively cultured on plates supplemented with 500 mg/L streptomycin.

Bacterial sweeps prepared from antrum and corpus biopsies during the same gastroscopy session [88] were thawed for re-culturing of *H. pylori* under standard conditions. Genomic DNA isolation and receptor binding analysis of these bacterial pools was performed as described in preceding sections.

### Fragment length analysis (FLA)

All fragment length analysis was performed at MWG Eurofins Medigenomix GmbH (Germany). FAM-labeled primers (see Table S2) were designed and optimized by using the Lasergene software (DNASTAR) and synthesized on site at MWG. Repetitive PCR-amplifications, with three different DNA polymerases (AmpliTaq Gold, Takara Taq, Bioline MyTaq), gave identical peak distribution at different dilutions, although with varying amplification strength (Fig. S4). For all FLA assays at least two dilutions with equal amounts of genomic DNA was used with comparable results.

### Electric mobility shift assay (EMSA)

Linear DNA containing the *sabA* promoter region (spanning −166 to +74) was generated by high-fidelity PCR using genomic DNA from SMI109 as template and primers *sabA-5* and *sabA-8*. Radio-labeled DNA fragments were generated by first pre-labeling the *sabA-5* primer using [γ^32^P]ATP (>3000 Ci/mmol; Perkin Elmer) and T4 kinase (Thermo Scientific). The binding reactions with 10 nM DNA and increasing concentrations of *E. coli* σ^70^-RNAP (Holoenzyme, Epicentre) were done as previously described [89]. The samples were separated on 4.5% Tris-Glycine (pH 8.5) polyarcylamide gel. The bands were visualized using Phosphor screen cassette, Typhoon scanner 9400 (GE Healthcare) and ImageJ software (NIH).

### Surface plasmon resonance analysis

Binding experiments shown in Fig. 4A and 4B were done with a Biacore3000 (GE Healthcare) at 25°C and analyzed with Scrubber 2 software (BioLogic Software). CM5 sensor chips where pre-coated with streptavidin (50 µg/ml) by injecting at 5 µl/min until approximately 5000 RU were obtained, using an amine coupling kit according to GE Healthcare protocol. This was followed by ethanolamine blockage and subsequent immobilization of 5′ biotin-labeled DNA fragments with running buffer [25 mM HEPES pH 7.5, 150 mM KCl, 10 mM MgCl_2_, 0.1 mM EDTA and 0.005% non-ionic surfactant polyoxyethylenesorbitan (P2O) (GE Healthcare)], by injecting at 5 µl/min to obtain approximately equal RU (<70) for every chip. All results were normalized to a T_13_ (wt) DNA-fragment used in all runs. DNA fragments were obtained by PCR amplification of genomic DNA from the A-, C-, or T-tract variants, using high fidelity DNA polymerase and 5′-Biotin-labeled primer paired with an unlabeled primer (Biotin-*sabA*-5 & *sabA*-8). 20 nM of the *E. coli* σ^70^-RNAP (Holoenzyme, Epicentre) was injected for 2 min at the rate of 100 µl/min. Between injections the σ^70^-RNAP was regenerated from the chip with a quick injection of 30 µl of 500 mM MgCl_2_. Binding to a DNA fragment of the *sabA* CDS was used as negative control, and subtracted from all data in Fig. 4. Experiments were performed in at least duplicates.

Binding experiments shown in Fig. 5D were done with the ProteOn system (Bio-Rad) at 25°C and analyzed with ProteOn manager 3.1 software (Bio-Rad). ProteOn GLC Sensor Chips were coated with streptavidin and blocked with ethanolamine, essentially as described in the preceding section. Biotinylated DNA fragments of the *sabA* promoter were immobilized by injection at 30 µl/min, to obtain approximately 120 RU for the two chips used. The *E. coli* σ^70^-RNAP (Holoenzyme, Epicentre) was injected in 5 different concentrations, 1.25, 2.5, 5, 10 and 20 ng/ml. The results were normalized to that of the T_13_ (wt) DNA-fragment.

### DNase I footprint assay

Binary complexes were formed by incubating 10 nM [γ^32^P]ATP-labeled DNA fragments and increasing concentrations of *E. coli* σ^70^-RNAP (Holoenzyme, Epicentre). Binding reactions were done in buffer B (25 mM HEPES pH 7.5, 0.1 mM EDTA, 5 mM DTT and 10% glycerol), 50 mM KCl and 0.5 mg/ml bovine serum albumin), for 30 min at 30°C. The reactions were subjected to DNase I digestion (0.3 U Recombinant DNase I, Ambion) and treated as previously described [90]. The samples were analyzed on a 6% denaturing polyacrylamide-8.3M urea gel. A+G Maxam Gilbert sequencing reactions of the same DNA fragments were loaded alongside the samples. The bands were visualized using Phosphor screen cassette and Typhoon scanner 9400 (GE Healthcare).

### Statistical analysis

We used the non-parametric two-tailed Mann-Whitney test for the statistical analyzes. Differences were considered significant when the *p* value was below 0.05. Significance levels are marked with *<0.05, **<0.01 and ***<0.005.

## Supporting Information

Figure S1 **The T-tract length affects** ***sabA*** **promoter activity.** A) Schematic illustration of the promoter DNA (−244 to +74) cloned in pRZ5202 to create the P*sabA*::*lacZ* transcriptional fusion plasmids assayed in Fig. S1B–C. See Materials and Methods for details. B) Promoter activity of P*sabA*::*lacZ* transcriptional fusion plasmids, containing *sabA* promoter from different *H. pylori* strains (see Fig. 1A–B and Table 1). β-galactosidase assays were performed in the *E. coli* strain AAG1, with cultures grown to OD_600_ of 2 and analyzed as described in Materials and Methods. C) Promoter activity of P*sabA*::*lacZ* transcriptional fusion plasmids, containing *sabA* promoter with different T-tract lengths (see Table 1). Samples were taken and β-galactosidase assays were performed as described in Fig. S1B.
(TIF)

Figure S2 **Highly conserved regions among** ***sabA*** **promoter sequences.** Alignment of 44 *sabA* promoter sequences from different *H. pylori* strains (see Table S1 for details). Marked by lines are the T-tract, −35 and −10 elements, and +1 transcriptional start site. The repetitive A-boxes located upstream of T-tract are boxed in red. Green and blue lines mark the distal and proximal UP-like elements. The promoter part that was deleted in the Δ_46_ variants (−94 to −49) is indicated by a dashed line. Stars (*) indicates >90% nucleotide conservation whereas black circle (•) indicates >75% conservation.
(TIF)

Figure S3 **Variability of T-tract length during growth** ***in vitro*** **.** A) Single clones, isolated from strain SMI109 after 3 months of passages on Brucella blood agar plates, were analyzed for binding to soluble ^125^I-sLex conjugate. The graph shows the percentage of binding of four high-binders (H), three low-binders (L), and a SMI109 *sabA* T_13_ and CT_8_-Off variant as a control. The length of the T-tract in each clone is shown above the bars. B) Single clones isolated from strain J99 analyzed for binding to soluble ^125^I-sLex conjugates. The experiment was performed as described in Fig. S3A. The graph shows binding of three low-binders (L), two high-binders (H), and a J99 T_19_ and CT_8_-Off variants as control.
(TIF)

Figure S4 **FLA assay optimization.** Fragment length analysis (FLA) was performed on defined mixes of genomic DNA prepared from the isogenic T_18_- and T_19_-variants of strain SMI109, using primers that amplified the *sabA* promoter (see Table S2). The percentage of genomic DNA used is stated to the left in the figure. Curves to the right show FLA-spectra after PCR-amplification using three different DNA polymerases, and genomic DNA from the T_18_-variant of SMI109 as template.
(TIF)

Figure S5 **Binding of RNA polymerase to P** ***sabA*** **DNA.** Electrophoretic mobility shift assay (EMSA) was used to analyze binding of *E. coli* σ^70^-RNAP to P*sabA* DNA. Left image: 10 nM of [γ-^32^P]ATP-labeled P*sabA* DNA (−166 to +74) was mixed with increasing concentrations of σ^70^-RNAP (0, 12.5, 25, 50 or 100 nM). Right diagram: 10 nM of [γ-^32^P]ATP-labeled P*sabA* DNA (−166 to +74), harboring different T-tract lengths, was mixed with 0 or 25 nM σ^70^-RNAP. The amount of shifted DNA, relative to buffer control, was calculated and plotted.
(TIF)

Figure S6 **RNA polymerase interacts with two UP-like elements in P** ***sabA*** **DNA.** A) Mapping of the binding site for σ^70^-RNAP to P*sabA* DNA lacking the proximal UP-like element (Δ_46_ variants, Fig. S2) using DNase I footprint assay. The assay was performed as described in Fig. 5B–C. The region protected from DNase I cleavage is marked by green line (distal UP-like element). Nucleotide positions, relative to the transcriptional start site, are shown to the right. B) Alignment of P*sabA* Δ_46_ DNA fragments (−166 to +74) analyzed as pdb structures in the Protean 3D software (Lasergene, DNASTAR). The same T-variants as analyzed in Fig. S6A was aligned to their respective wt P*sabA* DNA fragment. The image shows one view from a selected angle (Z-Y). The wt DNA fragments are displayed in black and Δ_46_ DNA fragment in light grey. C) *In silico* DNA structure predictions of P*sabA* (−166 to +74) with scrambled A-box analyzed as described in Fig. 6 and S6B. Images to the left show alignment of wt P*sabA* (black) and a variant with the closest A-box scrambled (orange), displayed in two orientations (Z-Y and Y-X). Middle images show DNA structure predictions of the P*sabA* (−166 to +74) harboring scrambled close A-box or UP-like elements, displayed in three dimensions. Right images show alignment of wt P*sabA* (black) and different scrambled UP-like elements (shades of grey), displayed in two orientations (Z-Y and Y-X).
(TIF)

Figure S7 **The nucleoid-associated proteins Hup and NapA do not influence SabA expression in** ***H. pylori*** **.** A) SMI109 wt, Δ*hup* and Δ*napA* strains were grown in Brucella broth at 37°C in 24-well plates under microaerophilic conditions. Growth was followed by OD_600_, and a minimum of 4 wells was analyzed at each time point, for each strain. B) RT-qPCR analysis of *sabA* mRNA levels in SMI109 wt, Δ*hup* and Δ*napA* strains, was performed as described in Fig. 1B. Samples were collected after growth on plate, as described in Fig. 1A, except for the Δ*hup* strain that was grown for 24 h longer (see Fig. S7A, and Materials and Methods for details). C) Analysis of SabA expression and sLex-receptor binding activity of the same set of strains as in Fig. S7B. The image shows one representative immunoblot with α-SabA antibodies, assayed as described in Fig. 1A and Fig. S10D. The bottom graph shows binding to soluble ^125^I-sLex-receptor conjugate. D) RT-qPCR analysis of *sabA* mRNA levels in wt and Δ*hup* derivatives of the T_13_- and T_18_-variants of SMI109, was performed as described in Fig. 1B. Samples were collected as described above and analyzed as described in Fig. 1B. E) Analysis of SabA expression and sLex-receptor binding activity of the same set of strains as in Fig. S7D. The image shows one representative immunoblot with α-SabA antibodies, assayed as described in Fig. 1A and Fig. S10E. The bottom graph shows binding to soluble ^125^I-sLex-receptor conjugate.
(TIF)

Figure S8 **Length variations of T- or A-tracts located adjacent to −35 elements in** ***H. pylori*** **genomes.** The 26695 genome was used to identify T- or A-tracts located adjacent to predicted −35 promoter elements (see Table 2 for complete list). Forty-five additional *H. pylori* genome sequences were downloaded from the NCBI server and used to analyze tract length variations in five selected loci; A) *hopD*, B) *hofA*, C) *hopM*, D) *hp_0350* and E) *sabB*. See Table S1 for more information.
(TIF)

Figure S9 ***hp_0350/pyrG*** **promoter sequence alignments.** *hp_0350*/*pyrG* promoter sequence alignments from 45 >different *Helicobacter* strains (see Table S1 for details). Marked by lines are the A-tract, −35 and −10 element, and +1 transcriptional start site. A-boxes located upstream of A-tract are boxed in red. Stars (*) indicates >90% nucleotide conservation whereas black circle (•) indicates >75% conservation.
(TIF)

Figure S10 **Normalization of SabA protein expression in different strains.** A) Analysis of SabA expression in a set of five *H. pylori* strains. Top images show one representative immunoblot analysis where the membrane was probed with α-SabA antibodies. Equal amounts of crude protein extracts were loaded in each lane as can be visualized in the PAGE Blue stained gel (lower image). Due to the difficulties of finding a protein that was not differentially expressed in the different strains, quantification of the PAGE Blue stained gel was used for normalization of the SabA expression values presented in Fig. 1A. B–E) Analysis of SabA expression in different variants of SMI109. Top image show one representative immunoblot analysis where the same membrane was probed with both α-SabA and α-AlpB antibodies. Equal amounts of crude protein extracts were loaded in each lane as can be visualized in the PAGE Blue stained gel (lower image). Expression of AlpB was used for normalization of the SabA expression values presented in (B) Fig. 2A, (C) Fig. 4C, (D) Fig. S7C and (E) Fig. S7E.
(TIF)

Table S1 **Length variations in repeat tracts of six relevant loci in different** ***Helicobacte*** **r genomes.**
(PDF)

Table S2 **Oligonucleotides used in this study.**
(PDF)
