# Supplementary figures and images for: A Repetitive DNA Element Regulates Expression of the Helicobacter pylori Sialic Acid Binding Adhesin by a Rheostat-like Mechanism
Source: PLoS Pathog. 2014 Jul 3;10(7):e1004234. doi: 10.1371/journal.ppat.1004234 (PMC4081817; doi:10.1371/journal.ppat.1004234)

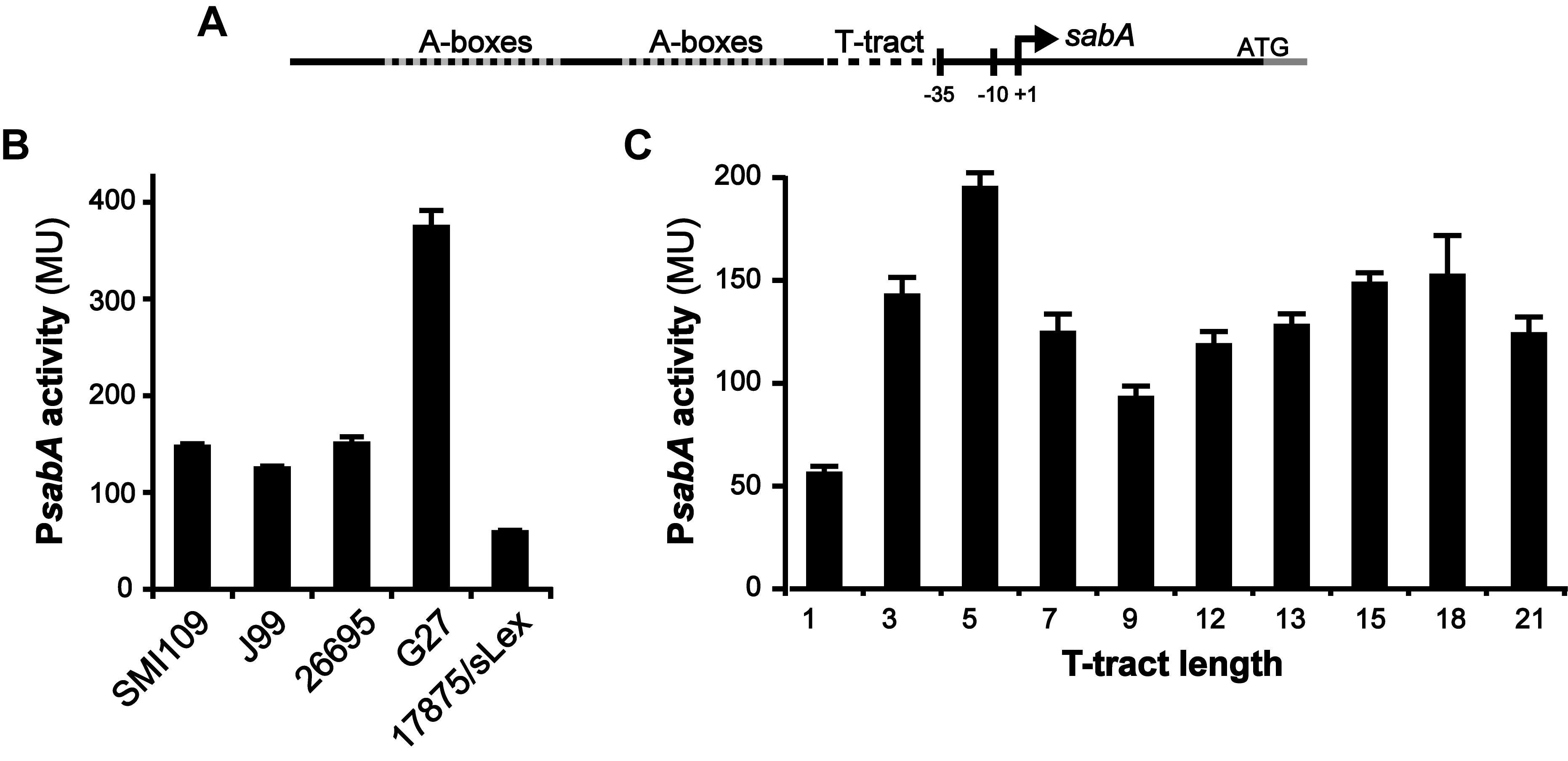

Supplement: Figure S1 — The T-tract length affects sabA promoter activity. A) Schematic illustration of the promoter DNA (−244 to +74) cloned in pRZ5202 to create the PsabA::lacZ transcriptional fusion plasmids assayed in Fig. S1B–C. See Materials and Methods for details. B) Promoter activity of PsabA::lacZ transcriptional fusion plasmids, containing sabA promoter from different H. pylori strains (see Fig. 1A–B and Table 1). β-galactosidase assays were performed in the E. coli strain AAG1, with cultures grown to OD600 of 2 and analyzed as described in Materials and Methods. C) Promoter activity of PsabA::lacZ transcriptional fusion plasmids, containing sabA promoter with different T-tract lengths (see Table 1). Samples were taken and β-galactosidase assays were performed as described in Fig. S1B. (TIF) [file ppat.1004234.s001.tif]

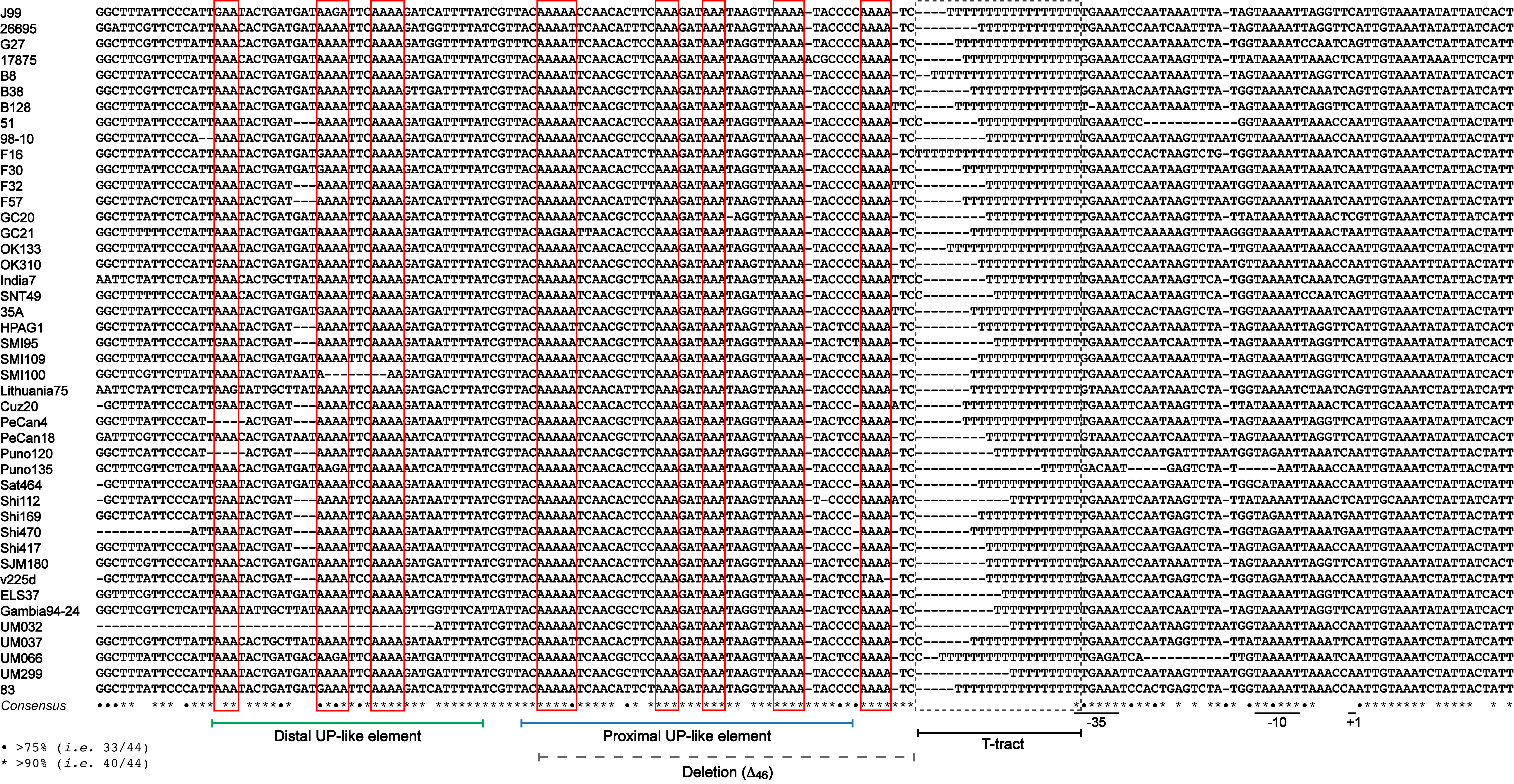

Supplement: Figure S2 — Highly conserved regions among sabA promoter sequences. Alignment of 44 sabA promoter sequences from different H. pylori strains (see Table S1 for details). Marked by lines are the T-tract, −35 and −10 elements, and +1 transcriptional start site. The repetitive A-boxes located upstream of T-tract are boxed in red. Green and blue lines mark the distal and proximal UP-like elements. The promoter part that was deleted in the Δ46 variants (−94 to −49) is indicated by a dashed line. Stars (*) indicates >90% nucleotide conservation whereas black circle (•) indicates >75% conservation. (TIF) [file ppat.1004234.s002.tif]

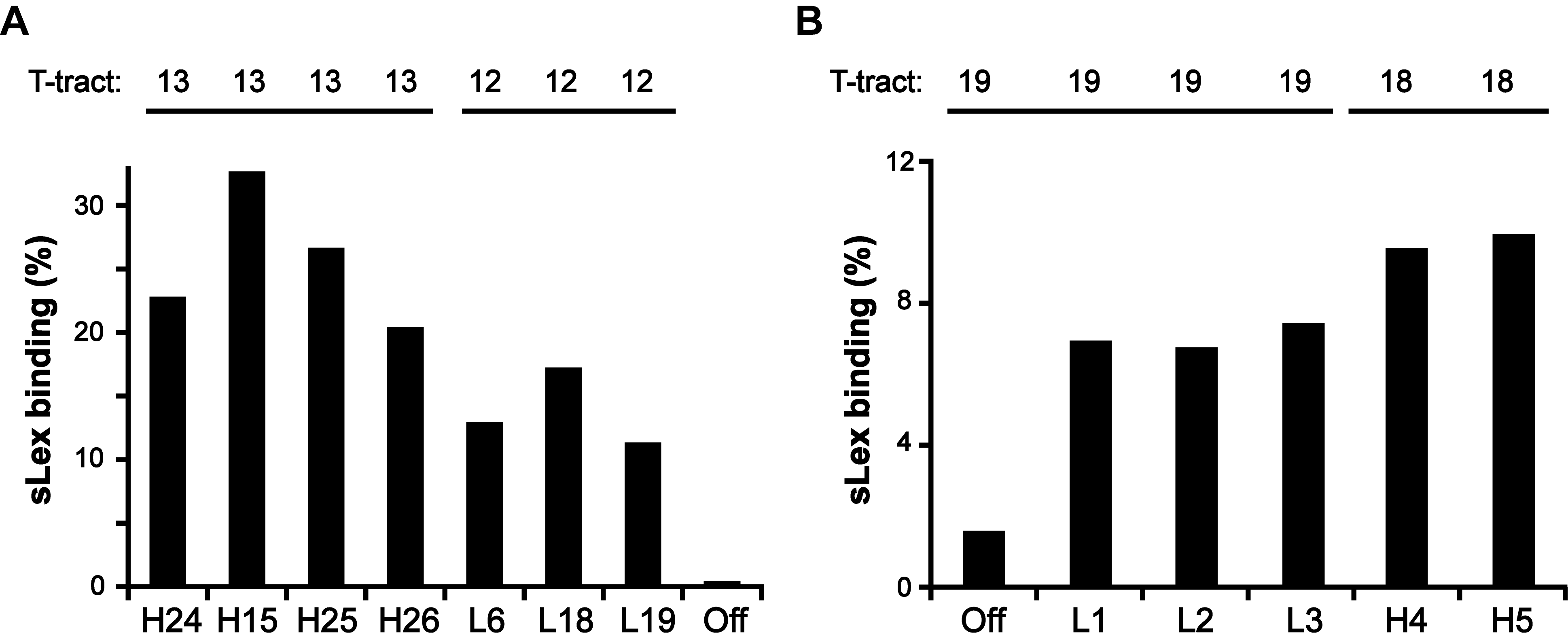

Supplement: Figure S3 — Variability of T-tract length during growth in vitro . A) Single clones, isolated from strain SMI109 after 3 months of passages on Brucella blood agar plates, were analyzed for binding to soluble 125I-sLex conjugate. The graph shows the percentage of binding of four high-binders (H), three low-binders (L), and a SMI109 sabA T13 and CT8-Off variant as a control. The length of the T-tract in each clone is shown above the bars. B) Single clones isolated from strain J99 analyzed for binding to soluble 125I-sLex conjugates. The experiment was performed as described in Fig. S3A. The graph shows binding of three low-binders (L), two high-binders (H), and a J99 T19 and CT8-Off variants as control. (TIF) [file ppat.1004234.s003.tif]

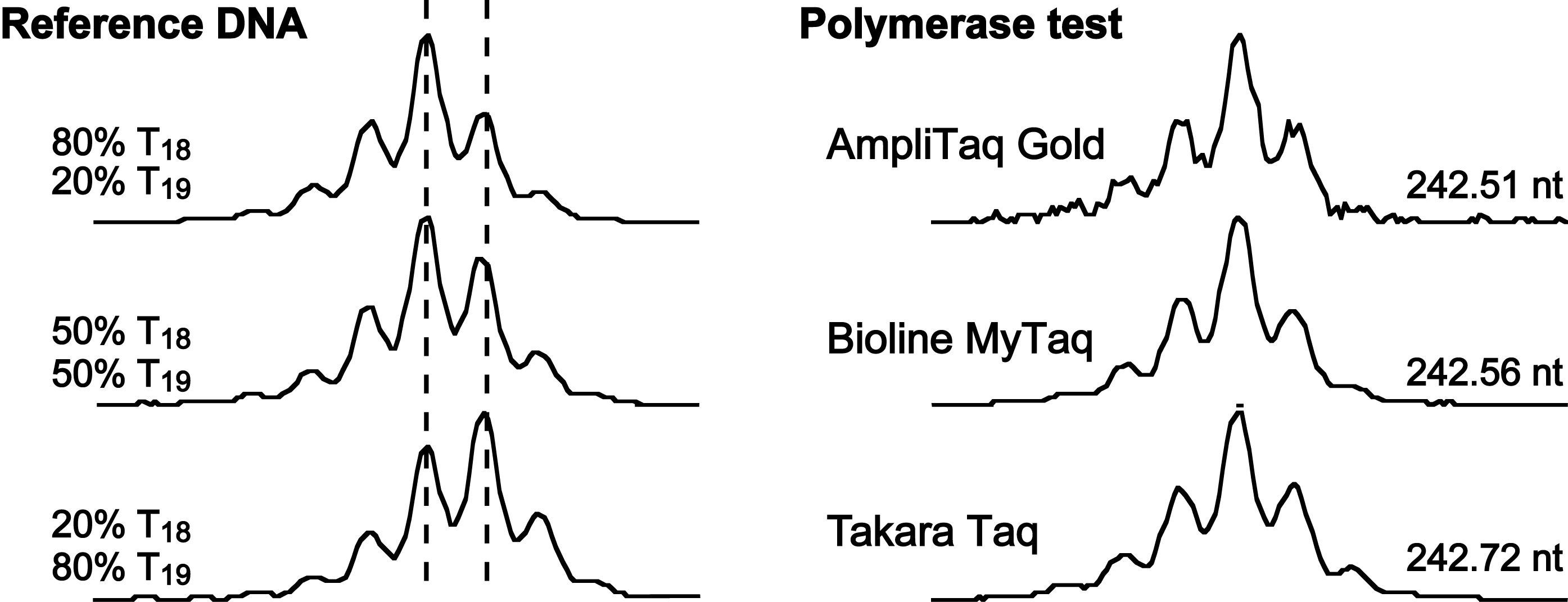

Supplement: Figure S4 — FLA assay optimization. Fragment length analysis (FLA) was performed on defined mixes of genomic DNA prepared from the isogenic T18- and T19-variants of strain SMI109, using primers that amplified the sabA promoter (see Table S2). The percentage of genomic DNA used is stated to the left in the figure. Curves to the right show FLA-spectra after PCR-amplification using three different DNA polymerases, and genomic DNA from the T18-variant of SMI109 as template. (TIF) [file ppat.1004234.s004.tif]

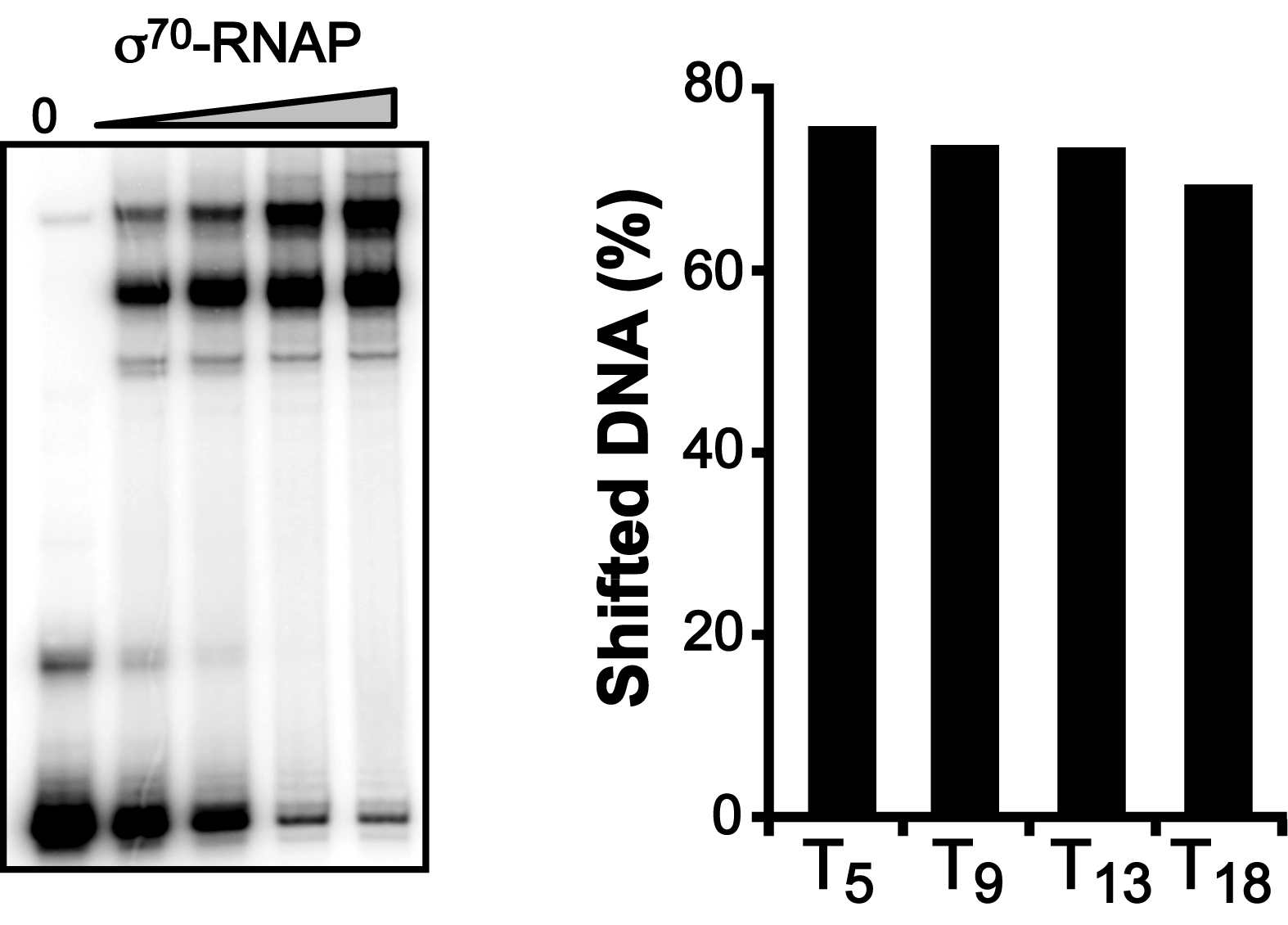

Supplement: Figure S5 — Binding of RNA polymerase to P sabA DNA. Electrophoretic mobility shift assay (EMSA) was used to analyze binding of E. coli σ70-RNAP to PsabA DNA. Left image: 10 nM of [γ-32P]ATP-labeled PsabA DNA (−166 to +74) was mixed with increasing concentrations of σ70-RNAP (0, 12.5, 25, 50 or 100 nM). Right diagram: 10 nM of [γ-32P]ATP-labeled PsabA DNA (−166 to +74), harboring different T-tract lengths, was mixed with 0 or 25 nM σ70-RNAP. The amount of shifted DNA, relative to buffer control, was calculated and plotted. (TIF) [file ppat.1004234.s005.tif]

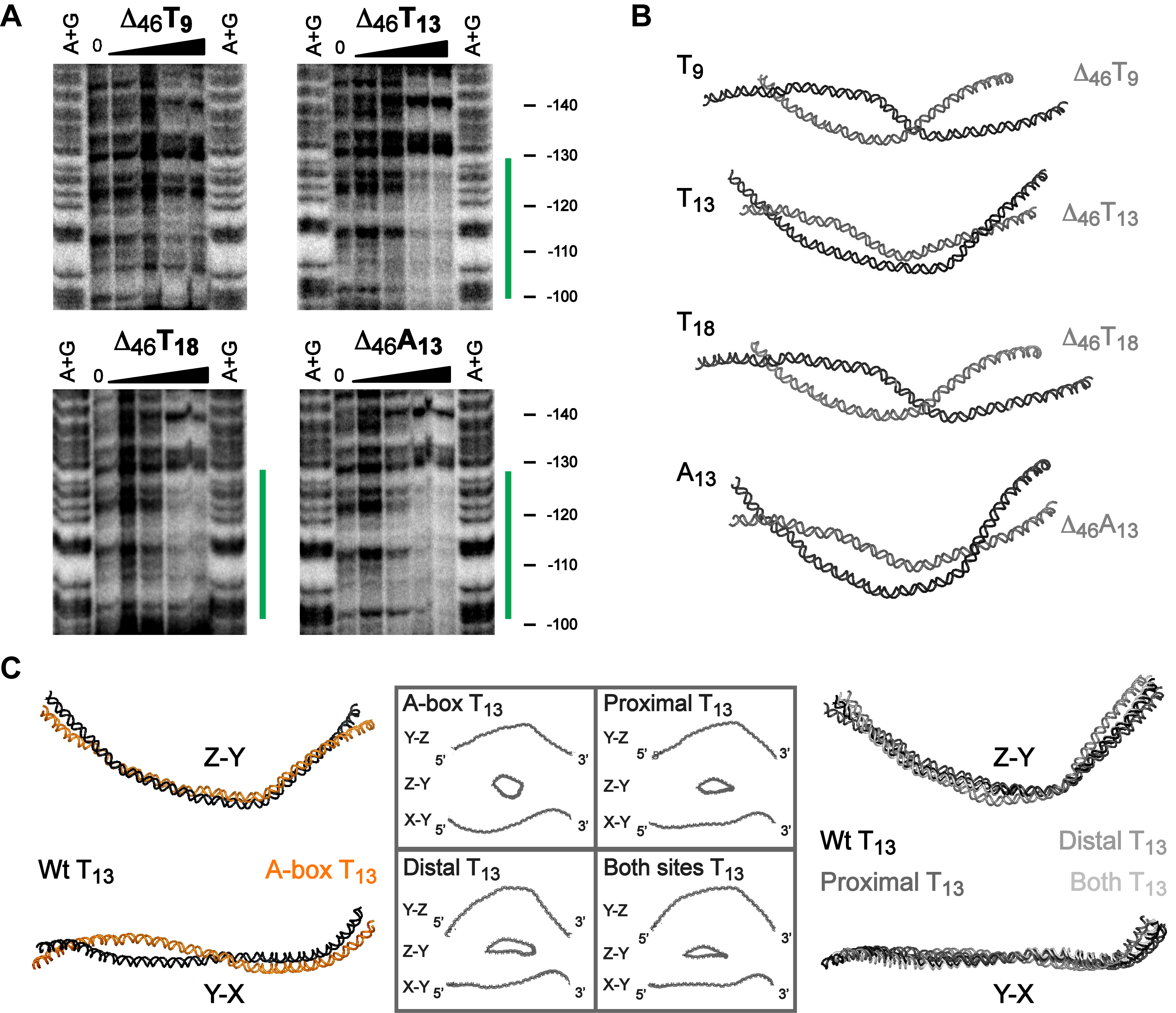

Supplement: Figure S6 — RNA polymerase interacts with two UP-like elements in P sabA DNA. A) Mapping of the binding site for σ70-RNAP to PsabA DNA lacking the proximal UP-like element (Δ46 variants, Fig. S2) using DNase I footprint assay. The assay was performed as described in Fig. 5B–C. The region protected from DNase I cleavage is marked by green line (distal UP-like element). Nucleotide positions, relative to the transcriptional start site, are shown to the right. B) Alignment of PsabA Δ46 DNA fragments (−166 to +74) analyzed as pdb structures in the Protean 3D software (Lasergene, DNASTAR). The same T-variants as analyzed in Fig. S6A was aligned to their respective wt PsabA DNA fragment. The image shows one view from a selected angle (Z-Y). The wt DNA fragments are displayed in black and Δ46 DNA fragment in light grey. C) In silico DNA structure predictions of PsabA (−166 to +74) with scrambled A-box analyzed as described in Fig. 6 and S6B. Images to the left show alignment of wt PsabA (black) and a variant with the closest A-box scrambled (orange), displayed in two orientations (Z-Y and Y-X). Middle images show DNA structure predictions of the PsabA (−166 to +74) harboring scrambled close A-box or UP-like elements, displayed in three dimensions. Right images show alignment of wt PsabA (black) and different scrambled UP-like elements (shades of grey), displayed in two orientations (Z-Y and Y-X). (TIF) [file ppat.1004234.s006.tif]

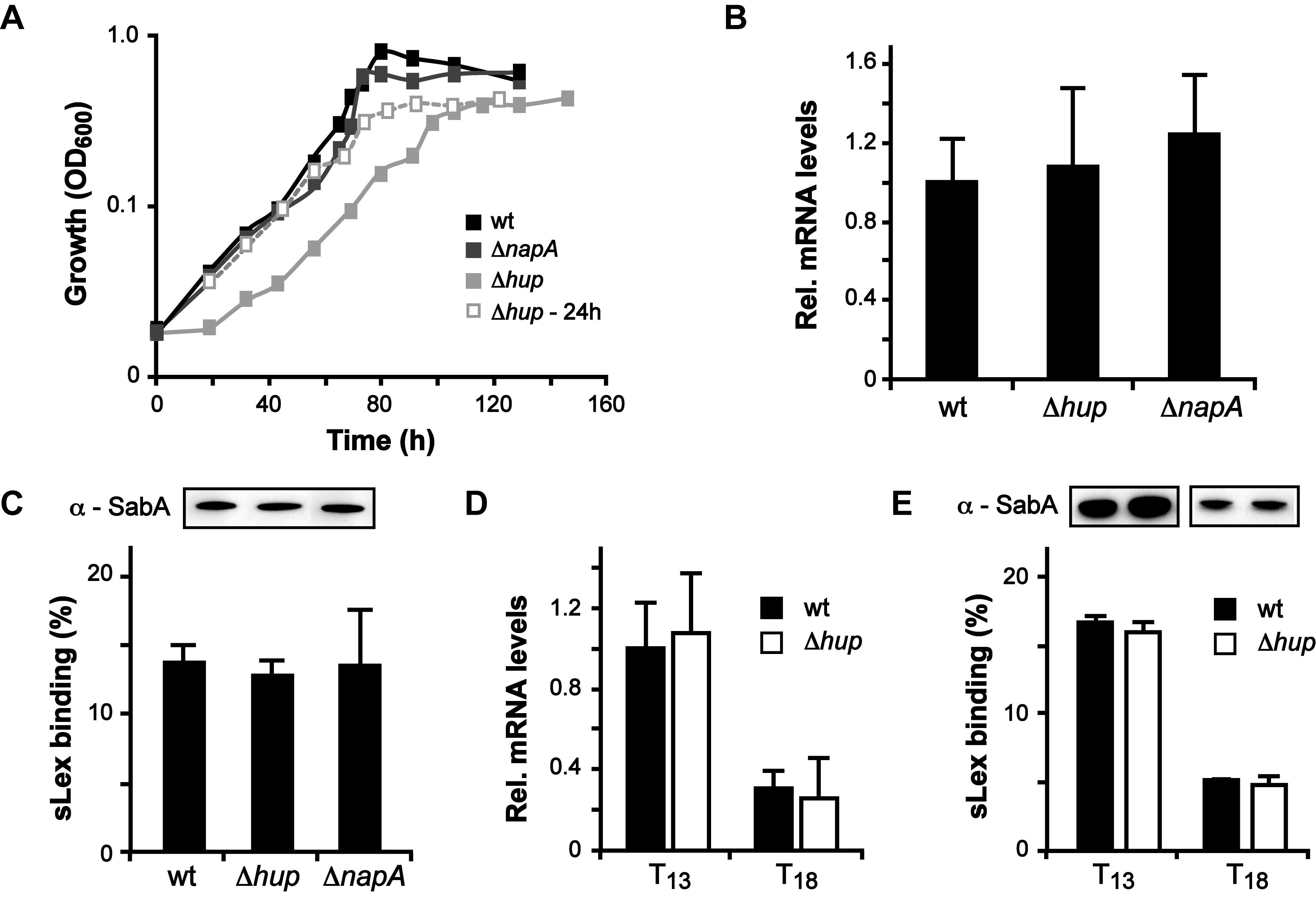

Supplement: Figure S7 — The nucleoid-associated proteins Hup and NapA do not influence SabA expression in H. pylori . A) SMI109 wt, Δhup and ΔnapA strains were grown in Brucella broth at 37°C in 24-well plates under microaerophilic conditions. Growth was followed by OD600, and a minimum of 4 wells was analyzed at each time point, for each strain. B) RT-qPCR analysis of sabA mRNA levels in SMI109 wt, Δhup and ΔnapA strains, was performed as described in Fig. 1B. Samples were collected after growth on plate, as described in Fig. 1A, except for the Δhup strain that was grown for 24 h longer (see Fig. S7A, and Materials and Methods for details). C) Analysis of SabA expression and sLex-receptor binding activity of the same set of strains as in Fig. S7B. The image shows one representative immunoblot with α-SabA antibodies, assayed as described in Fig. 1A and Fig. S10D. The bottom graph shows binding to soluble 125I-sLex-receptor conjugate. D) RT-qPCR analysis of sabA mRNA levels in wt and Δhup derivatives of the T13- and T18-variants of SMI109, was performed as described in Fig. 1B. Samples were collected as described above and analyzed as described in Fig. 1B. E) Analysis of SabA expression and sLex-receptor binding activity of the same set of strains as in Fig. S7D. The image shows one representative immunoblot with α-SabA antibodies, assayed as described in Fig. 1A and Fig. S10E. The bottom graph shows binding to soluble 125I-sLex-receptor conjugate. (TIF) [file ppat.1004234.s007.tif]

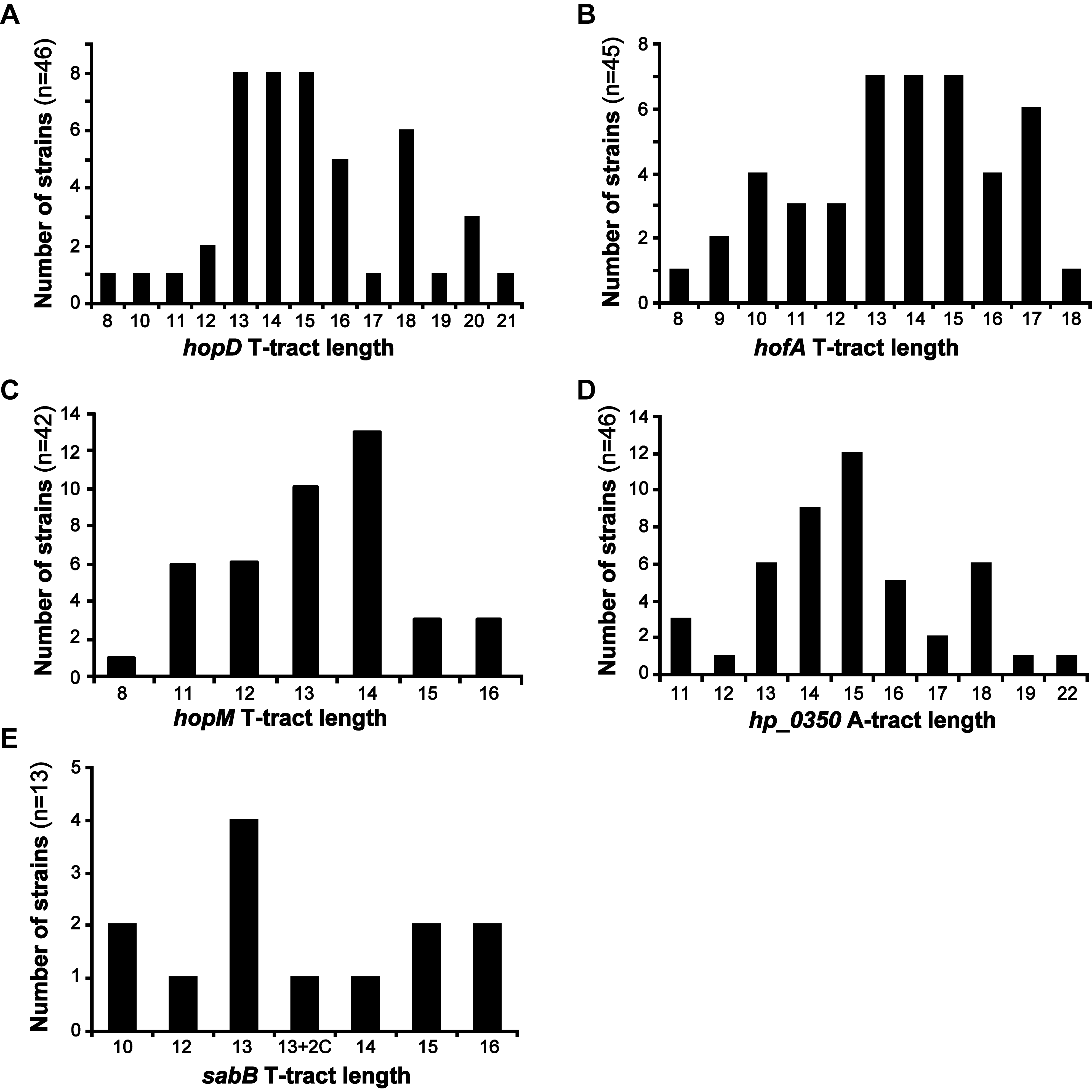

Supplement: Figure S8 — Length variations of T- or A-tracts located adjacent to −35 elements in H. pylori genomes. The 26695 genome was used to identify T- or A-tracts located adjacent to predicted −35 promoter elements (see Table 2 for complete list). Forty-five additional H. pylori genome sequences were downloaded from the NCBI server and used to analyze tract length variations in five selected loci; A) hopD, B) hofA, C) hopM, D) hp_0350 and E) sabB. See Table S1 for more information. (TIF) [file ppat.1004234.s008.tif]

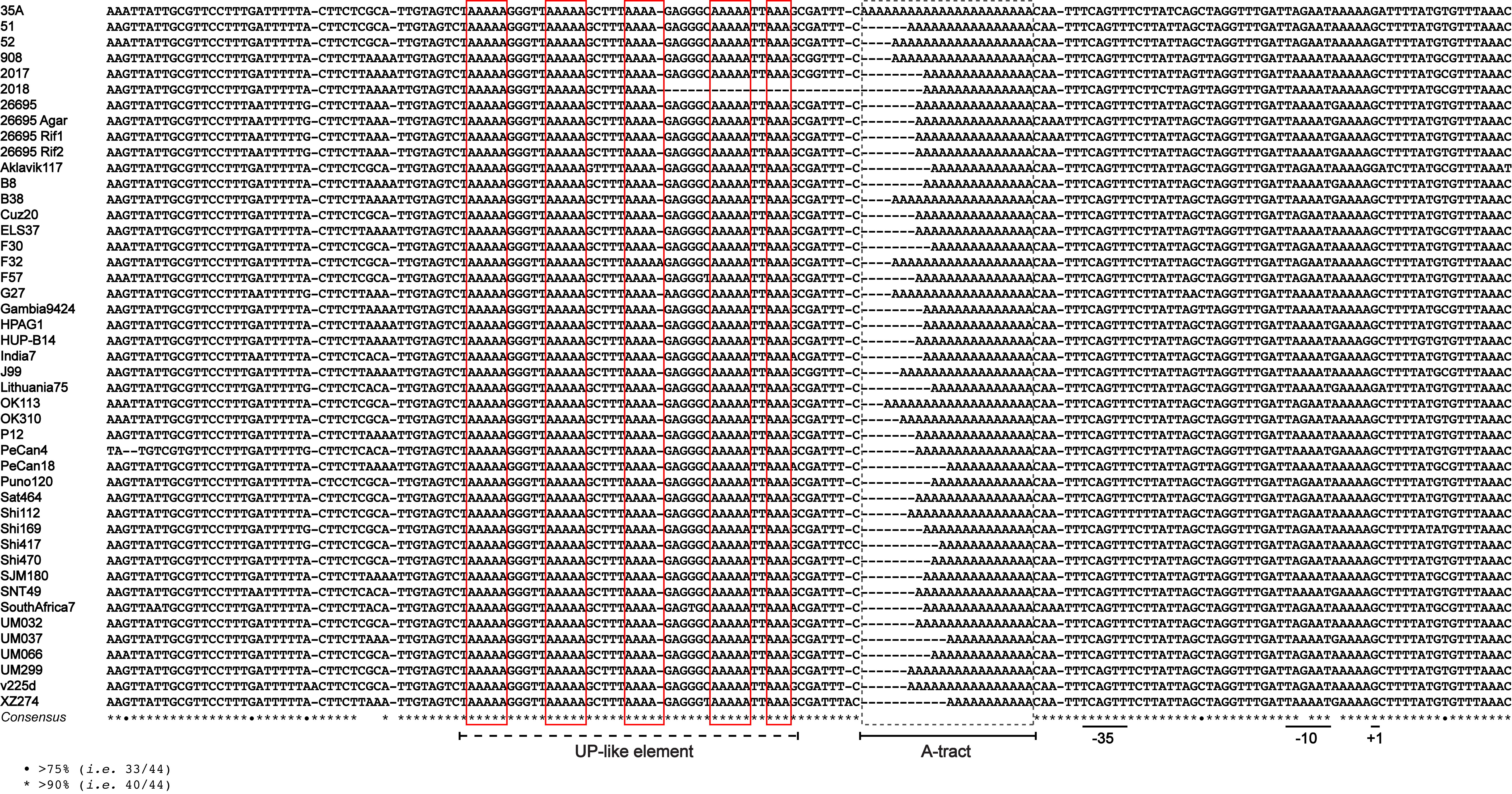

Supplement: Figure S9 — hp_0350/pyrG promoter sequence alignments. hp_0350/pyrG promoter sequence alignments from 45 >different Helicobacter strains (see Table S1 for details). Marked by lines are the A-tract, −35 and −10 element, and +1 transcriptional start site. A-boxes located upstream of A-tract are boxed in red. Stars (*) indicates >90% nucleotide conservation whereas black circle (•) indicates >75% conservation. (TIF) [file ppat.1004234.s009.tif]

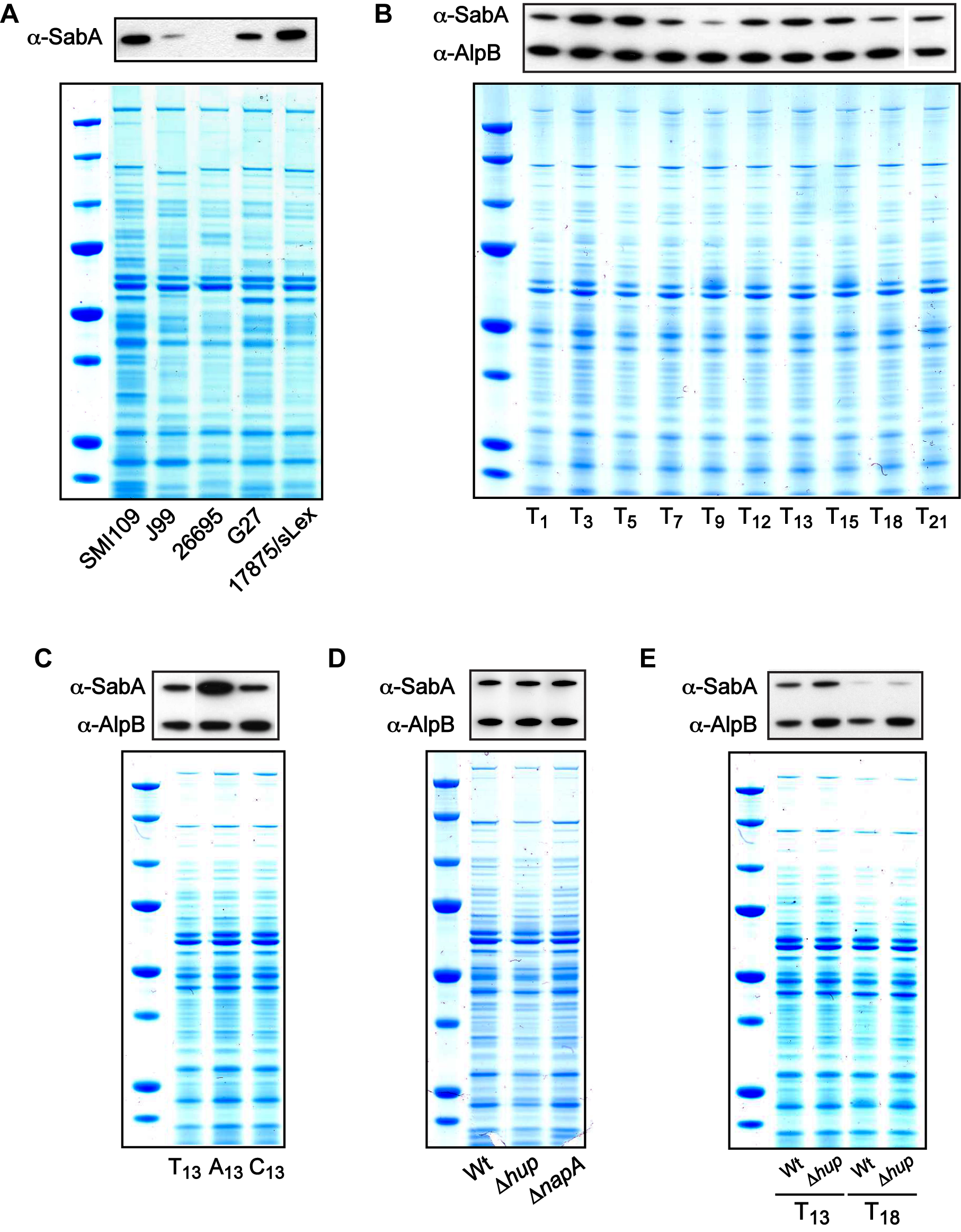

Supplement: Figure S10 — Normalization of SabA protein expression in different strains. A) Analysis of SabA expression in a set of five H. pylori strains. Top images show one representative immunoblot analysis where the membrane was probed with α-SabA antibodies. Equal amounts of crude protein extracts were loaded in each lane as can be visualized in the PAGE Blue stained gel (lower image). Due to the difficulties of finding a protein that was not differentially expressed in the different strains, quantification of the PAGE Blue stained gel was used for normalization of the SabA expression values presented in Fig. 1A. B–E) Analysis of SabA expression in different variants of SMI109. Top image show one representative immunoblot analysis where the same membrane was probed with both α-SabA and α-AlpB antibodies. Equal amounts of crude protein extracts were loaded in each lane as can be visualized in the PAGE Blue stained gel (lower image). Expression of AlpB was used for normalization of the SabA expression values presented in (B) Fig. 2A, (C) Fig. 4C, (D) Fig. S7C and (E) Fig. S7E. (TIF) [file ppat.1004234.s010.tif]
