# Supplementary material for: A Repetitive DNA Element Regulates Expression of the Helicobacter pylori Sialic Acid Binding Adhesin by a Rheostat-like Mechanism
Source: PLoS Pathog. 2014 Jul 3;10(7):e1004234. doi: 10.1371/journal.ppat.1004234 (PMC4081817; doi:10.1371/journal.ppat.1004234)
Supplement: Table S1 — Length variations in repeat tracts of six relevant loci in different Helicobacte r genomes. (PDF) [file ppat.1004234.s011.pdf]

Table S1. Lenght variations of repeat tracts of six relevant loci in different *Helicobacter* genomes.

Strain name	NCBI Ref Seq ID	*sabA*	*sabB*	*hopD*	*hofA*	*hopM**	*hp_0350*	
**Genome sequences**								
35A	NC_017360.1	15T	-	14T	14T	13T	22A	
51	NC_017382.1	14T	-	14T	15T	16T	16A	
52	NC_017354.1	-	13T	15T	12T	14T	18A	
83	NC_017375.1	17T	-	13T	15T	-	16A	
908	NC_017357.1	-	-	13T	14T	14T,	18A	
2017	NC_017374.1	-	-	18T	13T	fragmented	14A	
2018	NC_017381.1	-	-	15T	13T	gene	18A	
26695	NC_000915.1	14T	16T	15T	11T	14T	15A	Identical in all 26695 genomes
Aklavik86	NC_019563.1	-	-	14T	-	13T	15A	
Aklavik117	NC_019560.1	-	-	18T	14T	16T	13A	
B8	NC_014256.1	20T	-	15T	17T	14T	14A	
B38	NC_012973.1	14T	-	15T	14T	14T	18A	
Cuz20	NC_017358.1	16T	-	13T	13T	13T	15A	
ELS37	NC_017063.1	11T	12T	8T	12T	11T	15A	
F16	NC_017368.1	22T	-	17T	13T	13T	15A	
F30	NC_017365.1	16T	-	14T	10T	15T	13A	
F32	NC_017366.1	13T	-	20T	16T	14T	18A	
F57	NC_017367.1	16T	-	13T	14T	14T	15A	
G27	NC_011333.1	17T	15T	21T	16T	8T	18A	
Gambia9424	NC_017371.1	12T	-	18T	13T	13T	14A	
HPAG1	NC_008086.1	14T	13T	19T	18T	14T	14A	
HUP-B14	NC_017733.1	-	-	13T	17T	14T	15A	
India7	NC_017372.1	13T	-	10T	16T	13T	14A	
J99	NC_000921.1	18T	10T	14T	15T	13T	17A	
Lithuania75	NC_017362.1	14T	13T	15T	8T	11T	13A	
OK113	NC_020508.1	18T	-	20T	10T	14T	19A	
OK310	NC_020509.1	14T	-	15T	10T	14T	17A	
P12	NC_011498.1	-	16T	18T	17T	14T	15A	
PeCan4	NC_014555.1	16T	13T	16T	17T	13T	15A	
PeCan18	NC_017742.1	12T	11T & 2C	12T	11T	11T	11A	

Table S1. Lenght variations of repeat tracts of six relevant loci in different *Helicobacter* genomes.

Strain name	NCBI Ref Seq ID	*sabA*	*sabB*	*hopD*	*hofA*	*hopM**	*hp_0350*
Puno120	NC_017378.1	12T	-	13T	11T	11T	13A
Puno135	NC_017379.1	5T	-	14T	15T	11T	14A
Sat464	NC_017359.1	14T	-	16T	15T	12T	15A
Shi112	NC_017741.1	10T	-	12T	16T	14T	16A
Shi169	NC_017740.1	14T	-	18T	10T	12T	14A
Shi417	NC_017739.1	13T	-	14T	17T	13T	12A
Shi470	NC_010698.2	15T	-	13T	12T	11T	13A
SJM180	NC_014560.1	13T	15T	13T	14T	13T	14A
SNT49	NC_017376.1	12T	10T	18T	15T	14T	15A
SouthAfrica7	NC_017361.1	16T	-	16T	14T	12T	14A
UM032	NC_021215.2	10T	-	16T	13T	12T	15A
UM037	NC_021217.2	15T	-	16T	15T	16T	11A
UM066	NC_021218.2	19T	-	20T	17T	15T	13A
UM299	NC_021216.2	10T	-	15T	13T	12T	16A
v225d	NC_017355.1	13T	-	14T	9T	15T	16A
XZ274	NC_017926.1	-	14T	11T	9T	12T	11A

Sequencing of PCR products

B128	NZ_ABSY00000000.1	17T
98-10	NZ_ABSX00000000.1	13T
SMI12	na	18T
SMI52	na	10T
SMI63	na	15T
SMI66	na	17T
SMI95	na	12T
SMI100	na	17T
SMI109	KJ747959	13T
17875 sLex	na	15T
506	na	20T
508	na	20T
GC20	na	13T
GC21	na	17T

* This locus contained variable open reading frames (orf) in the different strains due to homologous recombination of homologous genes. Marked in grey are locus that contain a different orf than abbrivated in 26695.
hopM locus (HP_0227) contained *hopM* or *hopN* orfs

Table S1. Lenght variations of repeat tracts of six relevant loci in different *Helicobacter* genomes.

Strain name	NCBI Ref Seq ID	*sabA*	*sabB*	*hopD*	*hofA*	*hopM**	*hp_0350*
**Other *Helicobacter* species**							
Helicobacter acinonychis (cheetah) *	NC_008229.1		-	Similar 7T	-	-	15A
Helicobacter cetorum (dolphin) #	NC_017737.1		-	-	-	-	15A and 2T
Helicobacter cetorum (whale)	NC_017735.1		-	-	-	-	-
Helicobacter felis (cat)	NC_014810.2		-	-	-	-	-
Helicobacter mustelae (ferret)	NC_013949.1		-	-	-	-	-

* Eppinger M, Baar C, Linz B, Raddatz G, Lanz C et al. (2006). Who Ate Whom? Adaptive *Helicobacter* Genomic Changes That Accompanied a Host Jump from Early Humans to Large Felines. PLoS Genetics 2: e120

Kersulyte D, Rossi M, Berg DE (2013). Sequence Divergence and Conservation in Genomes of *Helicobacter cetorum* Strains from a Dolphin and a Whale. PLoS One 8:e83177
