# Supplementary material for: A Repetitive DNA Element Regulates Expression of the Helicobacter pylori Sialic Acid Binding Adhesin by a Rheostat-like Mechanism
Source: PLoS Pathog. 2014 Jul 3;10(7):e1004234. doi: 10.1371/journal.ppat.1004234 (PMC4081817; doi:10.1371/journal.ppat.1004234)
Supplement: Table S2 — Oligonucleotides used in this study. (PDF) [file ppat.1004234.s012.pdf]

Table S2. Oligonucleotides used in this study

Name	Sequence (5' to 3')
lacZ* fusion constructions in *E. coli* and *H. pylori	
sabA-1	ATGAATTCCTCTAGCAATGTGTGG
sabA-3	ATGGATCCCTTTTTCATAAAAGTGTTCCTT
sabA-Tf*	GGTTAAAATACTCCAAAATCTTTTTTTTTTTTGGAAATCCAATAAATT
sabA-Tr*	AATTTATTGGATTTCCAAAAAAAAAAAAGATTTTGGAGTATTTTAACC
P163-P165*, P167*	AAGATGATTTTATCGTTACTTTTTTTTTTTTTGGAAATCCAATAATTTATAGTA
P162	GTAACGATAAAATCATCTT
hypF R	GATCGTCGACGCCCCCTGTAACGAATA
hypF F	GATCGGATCCGAAAGAATTTTCAGCCAT
*pyrG*p F	GATCGTCGACGAAAGAATTTTCAGCCAT
*pyrG*p R	GATCGGATCCGCCCCCTGTAACGAATA
pyrG 9Tf	ACTGAAATTGTTTTTTTTTGAAATCGCTT
pyrG 9Tf	AAGCGATTTCAAAAAAAAACAATTTCAGT
Amut1-1F	GCTTCACAGATACATAGGTTACAGTACTC
Amut1-1R	GAGTACTGTAACCTATGTATCTGTGAAGC
Amut1-2F	TTATCGTTACACAGATCAACGCTTCACA
Amut1-2R	TGTGAAGCGTTGATCTGTGTAACGATAA
Amut2-1F	CTGATGATACAGTTCACAGGATGATTTTA
Amut2-1R	TAAAATCATCCTGTGAACTGTATCATCAG
Amut2-2F	GCTTTATTCCCATTACATACTGATGATAC
Amut2-2R	GTATCATCAGTATGTAATGGGAATAAAGC
**SMI109Δ*sabA*::*rpsLCAT* and T-variant constructions in *H. pylori***	
LA-F	AACCCCAAGCCCTCAAGATT
LA-R	GTGTTTAATCCATAGTTATAAAGCATCCTAAAACAAGAACGCATA
RA-F	CCCAGTTTGTCGCACTGATAAATGAAAAAGACAATTTTACTCT
RA-R	GGCAGTTGTCTATAATCGTTCTAA
rpsLCAT-F	TTTGTCAGCTATGCGTTCTTGTTTTAGGATGCTTTATAACTATGGATT
rpsLCAT-R	AGAGTAAAATTGTCTTTTTCATTTATCAGTGCGACAAACT
P93	AACCCCAAGCCCTCAAGATT
P96	GGCAGTTGTCTATAATCGTTCTAA
***hup* and *napA* deletion/insertion constructions**	
hup-1	CCCTTACCAAGCGATGGTGGGA
hup-2	TAGTGAAAGTGTATTACGCCACGC
hup-3Km	ACACAATCGCTCAAGACGTGTTCCGCTTTGTTCATGTGG
hup-4Km	CTAATTCCCATGTCAGCCGTGAGTGCCTAAATTCAAACCC
hup-5	GGGATTTTAATGGCTTGATTGTATCG
hup-in	CTCTTTTGCTGTTGTATTTACCCGC
napA1F	TTGATAATGGCAAGGAAGTGGA
napA1R	TCAAGCCATAGCGGATAAGCT
napA2F	CACGATCGCATCCGCTTGCA
napA2R	TTACCGTAACTTATGCGGACGAT
Kmup	CACGTCTTGAGCGATTGTGTAGG
Kmdn	ACGGCTGACATGGGAATTAGCCA
Km2	CGATTGTCTGTTGTGCCC

* Different variants of *sabA*-Tf and -Tr primers that harbor different length of the T tract were used. Here is one representative variant shown.

Table S2. Oligonucleotides used in this study

Name	Sequence (5' to 3')
**5' RACE and Primer extension**	
AB35	GATTGGGTGGTGCTGTTGTAGTTG
J99-8	GTTGTCTTCAGCGTGCAAGAGCG
sabA-8	CTTTTTCATAAAAGTGTTCCTT
**Sequencing *sabA* promoter**	
CL1	GCAATGTGTGGCAGCAAC
CL2	CTAGCGGGTTTTTGAGTATC
P67	TGGGTGGTGCTGTTGTAGTTGTT
P68	CATTTCATGGCGTTTGGTTTG
**EMSA, Footprint and Surface plasmon resonance**	
sabA-5	CGTTCTTGTTTTAGTTTGGC
Biotin-*sabA*-5	Biotin-CGTTCTTGTTTTAGTTTGGC
sabA-8	CTTTTTCATAAAAGTGTTCCTT
**FLA analysis – FAM primers**	
FAM-F	TATGCGTTCTTGTTTTAGTTT
FAM-Rlong	CTTTTTCATAAAATGTTCCTTAAAGTAAT
FAM-Rshort	ATTACTTTAAGGAACATTTTATGAAAAAG
**qPCR analysis**	
sabA-qP1F	TGAACGAAAAATACGAGCAA
sabA-qP1R	CCCGCATAAAGACTCCAAAA
sabA-qP2F	TTCGGTATTGGGTTTTTGGAG
sabA-qP2R	GCTTTAGTGGTAGCGTTTTGT
rrnA-2F	CTCCCTACGGGGGAAAGATT
rrnA-2R	GTAGGAGTCTGGACCGTGTC
ppk-2F	GCAAACCCAAAAAGGGAGTCA
ppk-2R	TGACCAATTCCCACAAATCCC
gyrA-1F	GCTAGGATCGTGGGTGATGT
gyrA-1R	TGGCTTCAGTGTAACGCATC
